# CyTOF workflow: differential discovery in high-throughput high-dimensional cytometry datasets

**DOI:** 10.12688/f1000research.11622.3

**Published:** 2019-05-24

**Authors:** Malgorzata Nowicka, Carsten Krieg, Helena L. Crowell, Lukas M. Weber, Felix J. Hartmann, Silvia Guglietta, Burkhard Becher, Mitchell P. Levesque, Mark D. Robinson

**Affiliations:** 1 Institute for Molecular Life Sciences, University of Zurich, Zurich, 8057, Switzerland; 2 SIB Swiss Institute of Bioinformatics, University of Zurich, Zurich, 8057, Switzerland; 3 Institute of Experimental Immunology, University of Zurich, Zurich, 8057, Switzerland; 4 Department of Experimental Oncology, European Institute of Oncology, Via Adamello 16, Milan, I-20139, Italy; 5 Department of Dermatology, University Hospital Zurich, Zurich, CH-8091, Switzerland

**Keywords:** CyTOF, flow cytometry, differential analysis

## Abstract

High-dimensional mass and flow cytometry (HDCyto) experiments have become a method of choice for high-throughput interrogation and characterization of cell populations. Here, we present an updated R-based pipeline for differential analyses of HDCyto data, largely based on Bioconductor packages. We computationally define cell populations using FlowSOM clustering, and facilitate an optional but reproducible strategy for manual merging of algorithm-generated clusters. Our workflow offers different analysis paths, including association of cell type abundance with a phenotype or changes in signalling markers within specific subpopulations, or differential analyses of aggregated signals. Importantly, the differential analyses we show are based on regression frameworks where the HDCyto data is the response; thus, we are able to model arbitrary experimental designs, such as those with batch effects, paired designs and so on. In particular, we apply generalized linear mixed models or linear mixed models to analyses of cell population abundance or cell-population-specific analyses of signaling markers, allowing overdispersion in cell count or aggregated signals across samples to be appropriately modeled. To support the formal statistical analyses, we encourage exploratory data analysis at every step, including quality control (e.g., multi-dimensional scaling plots), reporting of clustering results (dimensionality reduction, heatmaps with dendrograms) and differential analyses (e.g., plots of aggregated signals).

## Introduction

Flow cytometry and the more recently introduced CyTOF (cytometry by time-of-flight mass spectrometry or mass cytometry) are high-throughput technologies that measure protein abundance on the surface or within cells. In flow cytometry, antibodies are labeled with fluorescent dyes and fluorescence intensity is measured using lasers and photodetectors. CyTOF utilizes antibodies tagged with metal isotopes from the lanthanide series, which have favorable chemistry and do not occur in biological systems; abundances per cell are recorded with a time-of-flight mass spectrometer. In either case, fluorescence intensities (flow cytometry) or ion counts (mass cytometry) are assumed to be proportional to the expression level of the antibody-targeted antigens of interest.

Due to the differences in acquisition, further distinct characteristics should be noted. Conventional fluorophore-based flow cytometry is non-destructive and can be used to sort cells for further analysis. However, because of the spectral overlap between fluorophores, *compensation* of the data needs to be performed ^1^, which also limits the number of parameters that can be measured simultaneously. Thus, standard flow cytometry experiments measure 6–12 parameters with modern systems measuring up to 20 channels ^2^, while new developments (e.g., BD FACSymphony) promise to increase this capacity towards 50. Moreover, flow cytometry offers the highest throughput with tens of thousands of cells measured per second at relatively low operating costs per sample.

By using rare metal isotopes in CyTOF, cell autofluorescence can be avoided and spectral overlap is drastically reduced. However, the sensitivity of mass spectrometry results in the measurement of metal impurities and oxide formations, which need to be carefully considered in antibody panel design (e.g., through antibody concentrations and coupling of antibodies to neighboring metals). Leipold *et al.* recently commented that *minimal spillover does not equal no spillover* ^3^. Nonetheless, CyTOF offers a high dimension of parameters measured per cell, with current panels using ~40 parameters and the promise of up to 100. Throughput of CyTOF is lower, at the rate of hundreds of cells per second, and cells are destroyed during ionization.

The ability of flow cytometry and mass cytometry to analyze individual cells at high-throughput scales has resulted in a wide range of biological and medical applications. For example, immunophenotyping assays are used to detect and quantify cell populations of interest, to uncover new cell populations and compare abundance of cell populations between different conditions, for example between patient groups ^4^. Thus, it can be used as a biomarker discovery tool.

Various methodological approaches aim for biomarker discovery ^5^. A common strategy, which we will refer to throughout this workflow as the “classic” approach, is to first identify cell populations of interest by manual gating or automated clustering ^6, 7^. Second, using statistical tests, one can determine which of the cell subpopulations or protein markers are associated with a phenotype (e.g., clinical outcome) of interest. Typically, cell subpopulation abundance expressed as cluster cell counts or median marker expression would be used in the statistical model to relate to the sample-level phenotype.

Importantly, there are many alternatives to what we propose below, and several methods have emerged. For instance, *Citrus* ^8^ tackles the differential discovery problem by strong over-clustering of the cells, and by building a hierarchy of clusters from very specific to general ones. Using model selection and regularization techniques, clusters and markers that associate with the outcome are identified. A further machine learning approach, *CellCnn* ^9^, learns the representation of clusters that are associated with the considered phenotype by means of convolutional neural networks, which makes it particularly applicable to detecting discriminating rare cell populations. Another approach, *cydar* ^10^ performs differential abundance analysis on “hypersphere” counts, where hyperspheres are defined using all markers, and calculates differential tests using the the generalized linear modeling capabilities of *edgeR* ^11^.

However, there are tradeoffs to consider. *Citrus* performs feature selection but does not provide significance levels, such as p-values, for the strength of associations. Due to its computational requirements, *Citrus* cannot be run on entire mass cytometry datasets and one typically must analyze a subset of the data. The “filters” from *CellCnn* may identify one or more cell subsets that distinguish experimental groups, while these groups may not necessarily correspond to any of the canonical cell types, since they are learned with a data-driven approach. Since the hyperspheres from *cydar* are defined using all markers, interpretation of differential expression of specific markers (e.g., functional markers) within cell populations is difficult.

A noticeable distinction between the machine-learning approaches and our classical regression approach is the configuration of the model. *Citrus* and *CellCnn* model the patient response as a function of the measured HDCyto values, whereas the classical approach models the HDCyto data itself as the response, thus putting the distributional assumptions on the experimental HDCyto data. This carries the distinct advantage that covariates (e.g., age, gender, batch) can be included, together with finding associations of the phenotype to the predictors of interest (e.g., cell type abundance). Specifically, neither *Citrus* nor *CellCnn* are able to directly account for covariates, such as paired experiments or presence of batches. Another recent approach, mixed-effects association testing for single cells ( *MASC*) uses the same “reverse” association approach that we illustrate below ^12^. Recently, we have formalized and compared various regression approaches, resulting in the *diffcyt* package ^13^.

Within the classical approach, hybrid methods are certainly possible, where discovery of interesting cell populations is done with one algorithm, and quantifications or signal aggregations are modeled in standard regression frameworks. For instance, *CellCnn* provides p-values from a t-test or Mann-Whitney U-test conducted on the frequencies of previously detected cell populations. Some caution is warranted here, in terms of using data twice – so-called double dipping or circular analysis – and making claims about the statistical evidence of a change in abundance where initial analyses of the same data were used to discover subpopulations. This topic has been discussed with respect to clustering other types of single cell data and then inferring the markers of such populations ^14^; however, it is less clear how much clustering affects cross-sample inferences.

Step by step, this workflow presents differential discovery analyses assembled from a suite of tools and methods that, in our view, lead to a higher level of flexibility and robust, statistically-supported and interpretable results. Cell population identification is conducted by means of unsupervised clustering using the *FlowSOM* and *ConsensusClusterPlus* packages, which together were among the best performing clustering approaches for high-dimensional cytometry data ^15^. Notably, *FlowSOM* scales easily to millions of cells and thus no subsetting of the data is required.

To be able to analyze arbitrary experimental designs (e.g., batch effects, paired experiments, etc.), we show how to conduct differential analysis of cell population abundances using generalized linear mixed models (GLMM) and of marker intensities using linear models (LM) and linear mixed models (LMM). For both differential abundance and expression analysis, we use methods implemented in the *diffcyt* package ^13^. Internally, model fitting is performed with packages *lme4* and *stats*, and hypothesis testing with the *multcomp* package.

For visualization, we use new plotting functions from the *CATALYST* package that employ *ggplot2* as their graphical engine. Notably, *CATALYST* delivers a suite of useful visual representations of HDCyto data characteristics, such as an MDS (multidimensional scaling) plot of aggregated signal for exploring sample similarities. The obtained cell populations are visualized using dimension reduction techniques (e.g., UMAP via the *umap* package) and heatmaps (via the *ComplexHeatmap* package ^16^) to represent characteristics of the annotated cell populations and identified biomarkers. (Note that an alternative R implementation of the UMAP algorithm with additional functionality is also available in the *uwot* package.)

The workflow is intentionally not fully automatic. First, we strongly advocate for exploratory data analysis to get an understanding of data characteristics before formal statistical modeling. Second, the workflow involves an optional step where the user can manually merge and annotate clusters (see Cluster merging and annotation section) but in a way that is easily reproducible. The CyTOF data used here (see Data description section) is already preprocessed; i.e., the normalization and de-barcoding, as well as removal of doublets, debris and dead cells, were already performed; further details are available in the Data preprocessing section.

Notably, this workflow is equally applicable to flow or mass cytometry datasets, for which the preprocessing steps have already been performed. In addition, the workflow is modular and can be adapted as new algorithms or new knowledge about how to best use existing tools comes to light. Alternative clustering algorithms such as the popular PhenoGraph algorithm ^17^ (e.g., via the *Rphenograph* package), dimensionality reduction techniques, such as diffusion maps ^18^ via the *destiny* package ^19^, t-SNE via the *Rtsne* and SIMLR ^20^ via the *SIMLR* package could be inserted into the workflow.

***Note:*** *To cite this workflow, please refer to this F1000 article https://f1000research.com/articles/6-748*.

## Reproducibility

To generate reproducible results, we set random seeds in several steps of the workflow. However, the default methods for random number generation in R were updated in R version 3.6.0 (released in April 2019; see R News for details). Therefore, for consistency with earlier versions of the workflow, we use the function RNGversion() to use the random number generation methods from the previous version of R. Note that this step is not required when running a standard analysis on a new dataset; it is included here for reproducibility and backward compatibility only.

RNGversion("3.5.3")

## Data description

We use a subset of CyTOF data originating from Bodenmiller *et al.* ^21^ that was also used in the *Citrus* paper ^8^. In the original study, peripheral blood mononuclear cells (PBMCs) in unstimulated and after 11 different stimulation conditions were measured for 8 healthy donors. For each sample, expression of 10 cell surface markers and 14 signaling markers was recorded. We perform our analysis on samples from the reference and one stimulated condition where cells were crosslinked for 30 minutes with B cell receptor/Fc receptor known as BCR/FcR-XL, resulting in 16 samples in total (8 patients, unstimulated and stimulated for each).

The original data is available from the Cytobank report. The subset used here can be downloaded from the Citrus Cytobank repository (files with _BCR-XL.fcs or _Reference.fcs endings) or from the *HDCytoData* ^13^ package via Bodenmiller_BCR_XL_flowSet() (see Data import section).

In both the Bodenmiller *et al.* and *Citrus* manuscripts, the 10 lineage markers were used to identify cell subpopulations. These were then investigated for differences between reference and stimulated cell subpopulations separately for each of the 14 functional markers. The same strategy is used in this workflow; 10 lineage markers are used for cell clustering and 14 functional markers are tested for differential expression between the reference and BCR/FcR-XL stimulation. Even though differential analysis of cell abundance was not in the scope of the Bodenmiller *et al.* experiment, we present it here to highlight the generality of the discovery.

## Data preprocessing

Conventional flow cytometers and mass cytometers produce .fcs files that can be manually analyzed using programs such as FlowJo [TriStar] or Cytobank ^22^, or using R/Bioconductor packages, such as *flowWorkspace* ^23^ and *openCyto* ^24^. During this initial analysis step, dead cells are removed, compensation is checked and with simple two dimensional scatter plots (e.g., marker intensity versus time), marker expression patterns are checked. Often, modern experiments are barcoded in order to remove analytical biases due to individual sample variation or acquisition time. Preprocessing steps including normalization using bead standards ^25^, de-barcoding ^26^ and compensation can be completed with the *CATALYST* package ^27^, which also provides a Shiny app for interactive analysis. Of course, preprocessing steps can occur using custom scripts within R or outside of R (e.g., Normalizer ^25^).

## Data import

We recommend as standard practice to keep an independent record of all samples collected, with additional information about the experimental condition, including sample or patient identifiers, processing batch and so on. That is, we recommend having a trail of metadata for each experiment. In our example, the metadata file, PBMC8_metadata.xlsx, can be downloaded from the Robinson Lab server with the download.file() function. For the workflow, the user should place it in the current working directory ( getwd()). Here, we load it into R with the read_excel() function from the *readxl* package and save it into a variable called md, but other file types and interfaces to read them in are also possible.

The data frame md contains the following columns:

- file_name with names of the .fcs files corresponding to the reference (suffix “Reference”) and BCR/FcR-XL stimulation (suffix “BCR-XL”) samples,
- sample_id with shorter unique names for each sample containing information about conditions and patient IDs. These will be used to label samples throughout the entire workflow.
- condition describes whether samples originate from the reference ( Ref) or stimulated ( BCRXL) condition,
- patient_id defines the IDs of patients.

library(readxl)                                                
url <- "http://imlspenticton.uzh.ch/robinson_lab/cytofWorkflow"
md <- "PBMC8_metadata.xlsx"                                    
download.file(file.path(url, md), destfile = md, mode = "wb")  
md <- read_excel(md)                                           
head(data.frame(md))                                           

##                            file_name sample_id condition patient_id
## 1    PBMC8_30min_patient1_BCR-XL.fcs    BCRXL1     BCRXL   Patient1
## 2 PBMC8_30min_patient1_Reference.fcs      Ref1       Ref   Patient1
## 3    PBMC8_30min_patient2_BCR-XL.fcs    BCRXL2     BCRXL   Patient2
## 4 PBMC8_30min_patient2_Reference.fcs      Ref2       Ref   Patient2
## 5    PBMC8_30min_patient3_BCR-XL.fcs    BCRXL3     BCRXL   Patient3
## 6 PBMC8_30min_patient3_Reference.fcs      Ref3       Ref   Patient3

In our example, the data from the .fcs files listed in the metadata can be loaded from the *HDCytoData* package ^13^.

library(HDCytoData)                                                               
fs <- Bodenmiller_BCR_XL_flowSet()                                                

Alternatively, the files can be downloaded manually from the Citrus Cytobank repository and loaded into R as a flowSet using read.flowSet() from the *flowCore* package ^28^. Importantly, read.flowSet() and the underlying read.FCS() functions, by default, may transform the marker intensities and remove cells with extreme positive values. This behavior can be controlled with arguments transformation and truncate_max_range, respectively.

In our example, information about the panel is also available in a file called PBMC8_panel.xlsx, and can be downloaded from the Robinson Lab server and loaded into a variable called panel. It contains columns for Isotope and Metal that define the atomic mass number and the symbol of the chemical element conjugated to the antibody, respectively, and Antigen, which specifies the protein marker that was targeted; two additional columns specify whether a channel belongs to the lineage or functional type of marker.

The isotope, metal and antigen information that the instrument receives is also stored in the flowFrame (container for one sample) or flowSet (container for multiple samples) objects. One can type fs[[1]] to see the first flowFrame, which contains a table with columns name and desc. Their content can be retrieved with accessors pData(parameters(fs[[1]])). The variable name corresponds to the column names in the flowSet object, and can be viewed in R via colnames(fs).

It should be checked that elements from panel can be matched to their corresponding entries in the flowSet object. Specifically, the entries in panel$Antigen must have an equivalent in the desc columns of the flowFrame objects. In the following analysis, we will often use marker IDs as column names in the tables containing expression values. As a cautionary note, during object conversion from one type to another (e.g., in the creation of data.frame from a matrix), some characters (e.g., dashes) in the dimension names are replaced with dots, which may cause problems in matching. To avoid this problem, we will replace problematic characters (dashes with underscores; colons with dots) when organizing all data (measurement data, panel, and experimental metadata) into a so-called daFrame (Differential Analysis Frame; see below).

panel <- "PBMC8_panel_v3.xlsx"                                     
download.file(file.path(url, panel), destfile = panel, mode = "wb")
panel <- read_excel(panel)                                         
head(data.frame(panel))                                            


##      fcs_colname antigen marker_class
## 1 CD3(110:114)Dd     CD3         type
## 2  CD45(In115)Dd    CD45         type
## 3 pNFkB(Nd142)Dd   pNFkB        state
## 4  pp38(Nd144)Dd    pp38        state
## 5   CD4(Nd145)Dd     CD4         type
## 6  CD20(Sm147)Dd    CD20         type

# spot check that all panel columns are in the flowSet object
all(panel$fcs_colname %in% colnames(fs))

## [1] TRUE

## Data transformation

Usually, the raw marker intensities read by a cytometer have strongly skewed distributions with varying ranges of expression, thus making it difficult to distinguish between the negative and positive cell populations. It is common practice to transform CyTOF marker intensities using, for example, arcsinh (inverse hyperbolic sine) with cofactor 5 ^8, 29^ to make the distributions more symmetric and to map them to a comparable range of expression, which is important for clustering. A cofactor of 150 has been promoted for flow cytometry, but users are free to implement alternative transformations, some of which are available from the transform() function of the *flowCore* package. By default, the daFrame() constructor (see next section) arcsinh transforms marker expressions with a cofactor of 5.

As the ranges of marker intensities can vary substantially, for visualization, we apply another transformation that scales the expression of all markers to values between 0 and 1 using low (e.g., 1%) and high (e.g., 99%) percentiles as the boundary. This additional transformation of the arcsinh-transformed data can sometimes give better visual representation of relative differences in marker expression between annotated cell populations. However, all computations (differential testing, hierarchical clustering etc.) are still performed on arcsinh-transformed not scaled expressions. Whether scaled expression values should be plotted is specified with argument scale = TRUE or FALSE in the respective visualizations (e.g., plotExprHeatmap() and plotClusterHeatmap()).

## Data organization

We will store all data used and returned throughout differential analysis in an object of class daFrame from the *CATALYST* package. The daFrame requires the following inputs:

- x: a flowSet containing the raw measurement data, or a character string that specifies the path to a set of .fcs files.
- panel: a data.frame containing, for each marker, i) its column name in the input raw data, ii) its targeted protein markers, and, optionally, iii) its class (type, state, or none).
- md: a data.frame with columns describing the experimental design.

Argument cols_to_use specifies which columns (channels) to retain from the input data. By default, all measurement parameters will be kept ( cols_to_use = NULL). Here, we only keep the channels listed in panel.

It is important to carefully check whether variables are of the desired type (factor, numeric, character), since input methods may convert columns into different data types. This is taken care of by the daFrame() constructor. For the statistical modeling, we want to make the condition variable a factor with the reference ( Ref) being the reference level. The order of factor levels can be defined with the levels parameter of the factor function or via relevel().

As a final note, the daFrame() constructor requires the filenames listed in the md$file_name column to match those in the flowSet.

# specify levels for conditions & sample IDs to assure desired ordering 
md$condition <- factor(md$condition, levels = c("Ref", "BCRXL"))        
md$sample_id <- factor(md$sample_id,                                    
    levels = md$sample_id[order(md$condition)])                          
                                                                                                
# construct daFrame                                                     
daf <- daFrame(fs, panel, md, cols_to_use = panel$fcs_colname)          

## Diagnostic plots

We propose some quick checks to verify whether the data we analyze globally represents what we expect; for example, whether samples that are replicates of one condition are more similar and are distinct from samples from another condition. Another important check is to verify that marker expression distributions do not have any abnormalities such as having different ranges or distinct distributions for a subset of the samples. This could highlight problems with the sample collection or data acquisition, or batch effects that were unexpected. Depending on the situation, one can then consider removing problematic markers or samples from further analysis; in the case of batch effects, a covariate column could be added to the metadata table and used below in the statistical analyses.

**Figure 1.**
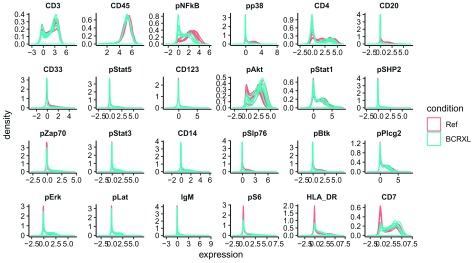
Per-sample smoothed densities of marker expression (arcsinh-transformed) of 10 lineage markers and 14 functional markers in the PBMC dataset. Two conditions: unstimulated (Ref) and stimulated with BCR/FcR-XL (BCRXL) for each of the 8 healthy donors are presented and colored by experimental condition.

The step below generates a plot with per-sample marker expression distributions, colored by condition ( Figure 1). Here, we can already see distinguishing markers, such as pNFkB and CD20, between stimulated and unstimulated conditions.

p <- plotExprs(daf, color_by = "condition")
p$facet$params$ncol <- 6                   
p                                          

**Figure 2.**
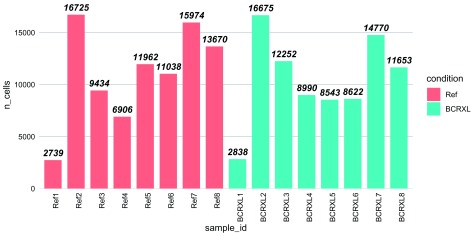
Barplot showing the number of cells measured for each sample in the PBMC dataset. Bars are colored by experimental condition: unstimulated (Ref) and stimulated with BCR/FcR-XL (BCRXL). Numbers in the names on the x-axis indicate patient IDs. Numbers on top of the bars indicate the cell counts.

Another spot check is the number of cells per sample ( Figure 2). This plot can be used as a guide together with other readouts to identify samples where not enough cells were assayed. The number of cells measured in each sample is also stored in the experiment_info slot of the daFrame’s metadata.

metadata(daf)$experiment_info$n_cells 

##  [1]  2838  2739 16675 16725 12252  9434  8990  6906  8543 11962  8622
## [12] 11038 14770 15974 11653 13670

plotCounts(daf, color_by = "condition")

## MDS plot

In transcriptomics applications, one of the most utilized exploratory plots is the multi-dimensional scaling (MDS) plot or a principal component analysis (PCA) plot. Such plots show similarities between samples measured in an unsupervised way and give a sense of how much differential expression can be detected before conducting any formal tests. In transcriptomics, distances between samples are calculated based on the expression of the top varying genes. We propose a similar plot for HDCyto data using median marker expression over all cells to calculate dissimilarities between samples (other aggregations are also possible, and one could reduce the number of top varying markers to include in the calculation). Ideally, samples should cluster well within the same condition, although this depends on the magnitude of the difference between experimental conditions. With this diagnostic, one can identify outlier samples and eliminate them if the circumstances warrant it. An MDS plot on the median marker expressions can be generated with plotMDS(), which internally calls the same-named *limma* function. 

**Figure 3.**
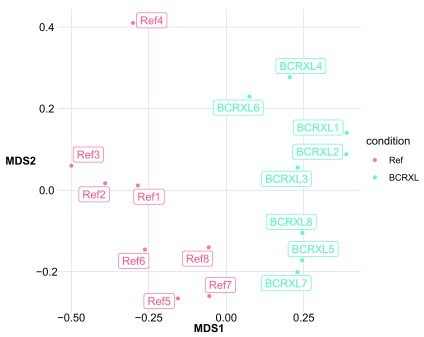
MDS plot for the unstimulated (Ref) and stimulated with BCR/FcR-XL (BCRXL) samples obtained for each of the 8 healthy donors in the PBMC dataset. Calculations are based on the median (arcsinh-transformed) marker expression of 10 lineage markers and 14 functional markers across all cells measured for each sample. Distances between samples in the plot approximate the typical change in medians. Numbers in the label names indicate patient IDs.

In our MDS plot on median marker expression values ( Figure 3), we can also see that the first dimension (MDS1) separates the unstimulated and stimulated samples reasonably well. The second dimension (MDS2) represents, to some degree, differences between patients. Most of the samples that originate from the same patient are placed at a similar point along the y-axis, for example, samples from patients 7, 5, and 8 are at the bottom of the plot, while samples from patient 4 are located at the top of the plot. This also indicates that the marker expression of individual patients is driving similarity and perhaps should be formally accounted for in the downstream statistical modeling.

plotMDS(daf, color_by = "condition")

**Figure 4.**
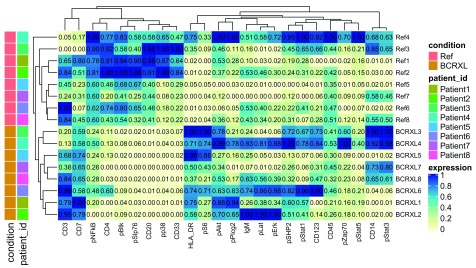
Heatmap of the median (arcsinh-transformed) marker expression of 10 lineage markers and 14 functional markers across all cells measured for each sample in the PBMC dataset. Color-coded with yellow for lower expression and blue for higher expression. The numbers in the heatmap represent the actual expression values. Dendrograms present clustering of samples (rows) and markers (columns) which is based on hierarchical clustering with Euclidean distance metric and average linkage. Row annotations on the left of the heatmap represent the two conditions: unstimulated (Ref) and stimulated with BCR/FcR-XL (BCRXL), and patient IDs for each of the 8 healthy donors.

In contrast to genomic applications, the number of variables measured for each sample is much lower in HDCyto data. In the former, thousands of genes are surveyed, whereas in the latter, ~20-50 antigens are typically targeted. Similar to the MDS plot above, a heatmap of the same data also gives insight into the structure of the data. The heatmap shows median marker intensities with clustered columns (markers) and rows (samples). We have used hierarchical clustering with average linkage and Euclidean distance, but also Ward’s linkage could be used ^8^, and in CyTOF applications, a cosine distance shows good performance ^30^. In this plot, we can see which markers drive the observed clustering of samples ( Figure 4).

As with the MDS plot, the dendrogram separates the reference and stimulated samples very well. Also, similar groupings of patients within experimental conditions are observed.

plotExprHeatmap(daf, bin_anno = TRUE, row_anno = TRUE)

## Marker ranking based on the non-redundancy score

In this step, we identify the ability of markers to explain the variance observed in each sample. In particular, we calculate the PCA-based non-redundancy score (NRS) ^17^. Markers with higher score explain a larger portion of variability present in a given sample.

**Figure 5.**
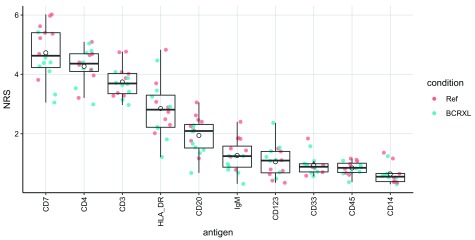
Non-redundancy scores for each of the 10 lineage markers and all samples in the PBMC dataset. The full points represent the per-sample NR scores (colored by experimental conditions), while empty black circles indicate the mean NR scores from all the samples. Markers on the x-axis are sorted according to the decreasing average NRS.

The average NRS can be used to select a subset of markers that are non-redundant in each sample but at the same time capture the overall diversity between samples. Such a subset of markers can then be used for cell population identification analysis (i.e., clustering). We note that there is no precise rule on how to choose the right cutoff for marker inclusion, but one option is to select a suitable number of the top-scoring markers. The number can be chosen by analyzing the plot with the NR scores ( Figure 5), where the markers are sorted by the decreasing average NRS. Based on prior biological knowledge, one can refine the marker selection and remove markers that are not likely to distinguish cell populations of interest, even if they have high scores, and add in markers with low scores but known to be important in discerning cell subgroups ^17^. Thus, the NRS analysis serves more as a guide to marker selection and is not meant as a hard rule.

In the dataset considered here ^8, 21^, we want to use all the 10 lineage markers, so there is no explicit need to restrict the set of cell surface markers, and the NRS serve as another quality control step. There may be other situations where this feature selection step would be of interest, for example, in panel design ^17^.

plotNRS(daf, markers = type_markers(daf), color_by = "condition")

## Cell population identification with FlowSOM and ConsensusClusterPlus

Cell population identification typically has been carried out by manual gating, a method based on visual inspection of a series of two-dimensional scatterplots. At each step, a subset of cells, either positive or negative for the two visualized markers, is selected and further stratified in the subsequent iterations until populations of interest across a range of marker combinations are captured. However, manual gating has drawbacks, such as subjectivity, bias toward well-known cell types, and inefficiency when analyzing large datasets, which also contribute to a lack of reproducibility ^5^.

Considerable effort has been made to improve and automate cell population identification, such as unsupervised clustering ^31^. However, not all methods scale well in terms of performance and speed from the lower dimensionality flow cytometry data to the higher dimensionality mass cytometry data ^15^, since clustering in higher dimensions can suffer the “curse of dimensionality”.

Beside the mathematical and algorithmic challenges of clustering, cell population identification may be difficult due to the chemical and biological aspects of the cytometry experiment itself. Therefore, caution should be taken when designing panels aimed at detecting rare cell populations by assigning higher sensitivity metals to rare markers. The right choice of a marker panel used for clustering can also be important. For example, it should include all markers that are relevant for cell type identification.

In this workflow, we conduct cell clustering with *FlowSOM* ^32^ and *ConsensusClusterPlus* ^33^, which appeared amongst the fastest and best performing clustering approaches in a recent study of HDCyto datasets ^15^. This ensemble showed strong performance in detecting both high and low frequency cell populations and is one of the fastest methods to run, which enables its interactive usage. We use a slight modification of the original workflow presented in the *FlowSOM* vignette, which we find more flexible. In particular, we directly call the ConsensusClusterPlus() function that is embedded in metaClustering_consensus(). Thus, we are able to access all the functionality of the *ConsensusClusterPlus* package to explore the number of clusters.

The *FlowSOM* workflow consists of three steps: i) building a self-organizing map (SOM), where cells are assigned according to their similarities to 100 (by default) grid points (or, so-called codebook vectors or codes) of the SOM; ii) building a minimal spanning tree, which is mainly used for graphical representation of the clusters, is skipped in this pipeline; and iii) *metaclustering* of the SOM codes, is performed with the *ConsensusClusterPlus* package. These are wrapped in the *CATALYST* function cluster(). Additionally, we add an optional round of manual expert-based merging of the metaclusters and allow this to be done in a reproducible fashion.

It is important to point out that we cluster all cells from all samples together. This strategy is beneficial, since we directly obtain cluster assignment for each cell, we label cell populations only once and the mapping of cell types between samples is automatically consistent. For a list of alternative approaches and their advantages and disadvantages, please refer to the Discussion section, where we consider: clustering per sample, clustering of data from different measurement batches and down-sampling in case of widely varying numbers of cells per sample.

*CATALYST* provides the wrapper function cluster() to perform both *FlowSOM* clustering and *ConsensusClusterPlus* metaclustering. The clustering IDs obtained after the first high-dimensional clustering step are added to the input daFrame’s rowData in the cluster_id column. The cluster codes for the lower dimensional metaclusterings to 2 through maxK clusters are stored as list element cluster_codes in the metadata. In this way, all levels of clustering are computed once and kept accessible for further investigation, visualization, and differential analysis.

The subset of markers to use for clustering is specified with argument cols_to_use. For future reference, the specified markers will be assigned class "type", and the remainder of markers will be assigned to be "state" markers. The sets of type and state markers can be accessed at any point with the type_markers() and state_markers() accessor functions, respectively.

In our example, we have specified marker classes in the input panel, and cluster() will default to using "type" markers for clustering. For clarity, we specify this explicitly via cols_to_use = type_markers(daf). We call ConsensusClusterPlus() with maximum number of clusters maxK = 20.

*FlowSOM* output can be sensitive to random starts ^15^. To make results reproducible, cluster() takes a seed argument that is passed to set.seed for random number generation, prior to calling BuildSOM(). It is advisable to rerun analyses with multiple random seeds, for two reasons. First, one can see how robust the detected clusters are, and second, when the goal is to find smaller cell populations, it may happen that, in some runs, random starting points do not represent rare cell populations, as the chance of selecting starting cells from them is low and they are merged into a larger cluster.

daf <- cluster(daf, cols_to_use = type_markers(daf), 
    xdim = 10, ydim = 10, maxK = 20, seed = 1234)    

Automatic approaches for selecting the number of clusters in HDCyto data do not always succeed ^15^. In general, we therefore recommend some level of over-clustering, and if desired, manual merging of clusters. Such a hierarchical approach is especially suited when the goal is to detect smaller cell populations.

The SPADE clustering analysis performed by Bodenmiller *et al.* ^21^ identified 6 main cell types (T-cells, monocytes, dendritic cells, B-cells, NK cells and surface- cells) that were further stratified into 14 more specific subpopulations (CD4+ T-cells, CD8+ T-cells, CD14+ HLA-DR high monocytes, CD14+ HLA-DR med monocytes, CD14+ HLA-DR low monocytes, CD14- HLA-DR high monocytes, CD14- HLA-DR med monocytes, CD14- HLA-DR low monocytes, dendritic cells, IgM+ B-cells, IgM- B-cells, NK cells, surface- CD14+ cells and surface- CD14- cells). In our analysis, we are interested in identifying the 6 main PBMC populations, including: CD4+ T-cells, CD8+ T-cells, monocytes, dendritic cells, NK cells and B-cells. Following the concept of over-clustering, we perform the metaclustering of the (by default) 100 SOM codes into more than expected number of groups. For example, stratification into 20 groups should give enough resolution to detect these main clusters. We can explore the clustering in a wide variety of visualizations: UMAP plots, heatmaps and the “delta area” from *ConsensusClusterPlus*.

When the interest is in studying more specific subpopulations at higher detail, one can follow a strategy of reclustering as described in the Obtaining higher resolution section, where we propose to repeat the workflow (clustering and differential analyses) after gating out a selected subpopulation (e.g., one of the large populations).

**Figure 6.**
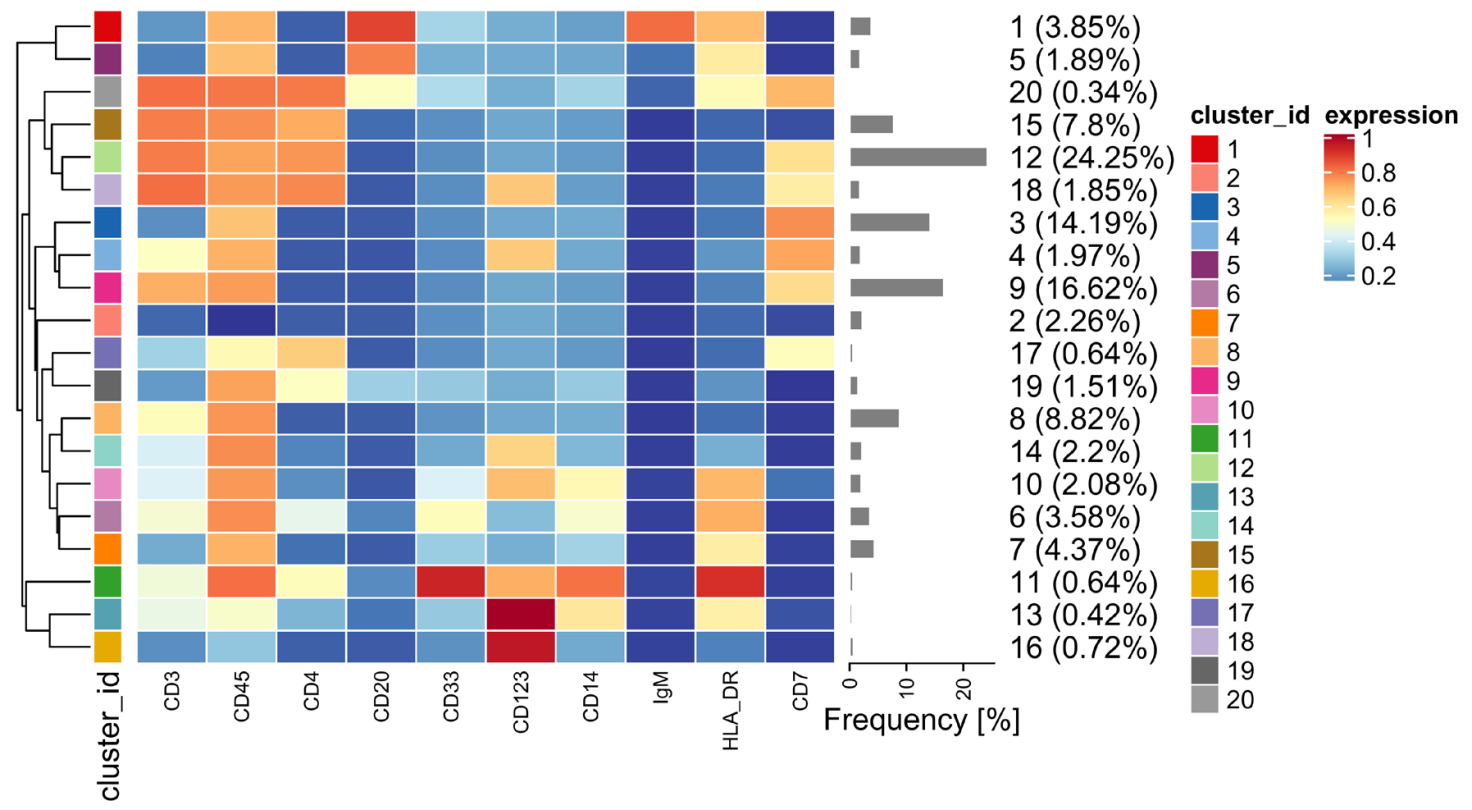
Heatmap of the median marker intensities of the 10 lineage markers across the 20 cell populations obtained with FlowSOM after the metaclustering step with ConsensusClusterPlus (PBMC data). The color in the heatmap represents the median of the arcsinh, 0-1 transformed marker expression calculated over cells from all the samples, varying from blue for lower expression to red for higher expression. The dendrogram on the left represents the hierarchical similarity between the 20 metaclusters (metric: Euclidean distance; linkage: average). Each cluster has a unique color assigned (bar on the left) which is identical in other visualizations of these 20 clusters (e.g., the UMAP shown in Figure 10) facilitating the figure interpretation. Barplot along the rows (clusters) and values in brackets on the right indicate the relative sizes of clusters.

We can then investigate characteristics of identified clusters with heatmaps that illustrate median marker expression in each cluster ( Figure 6). As the range of marker expression can vary substantially from marker to marker, we use the 0-1 transformed data for some visualizations (argument scale = TRUE in the respective plotting functions). However, to stay consistent with *FlowSOM* and *ConsensusClusterPlus*, we use the (arcsinh-transformed) unscaled data to generate the dendrogram of the hierarchical structure of metaclusters.

**Figure 7.**
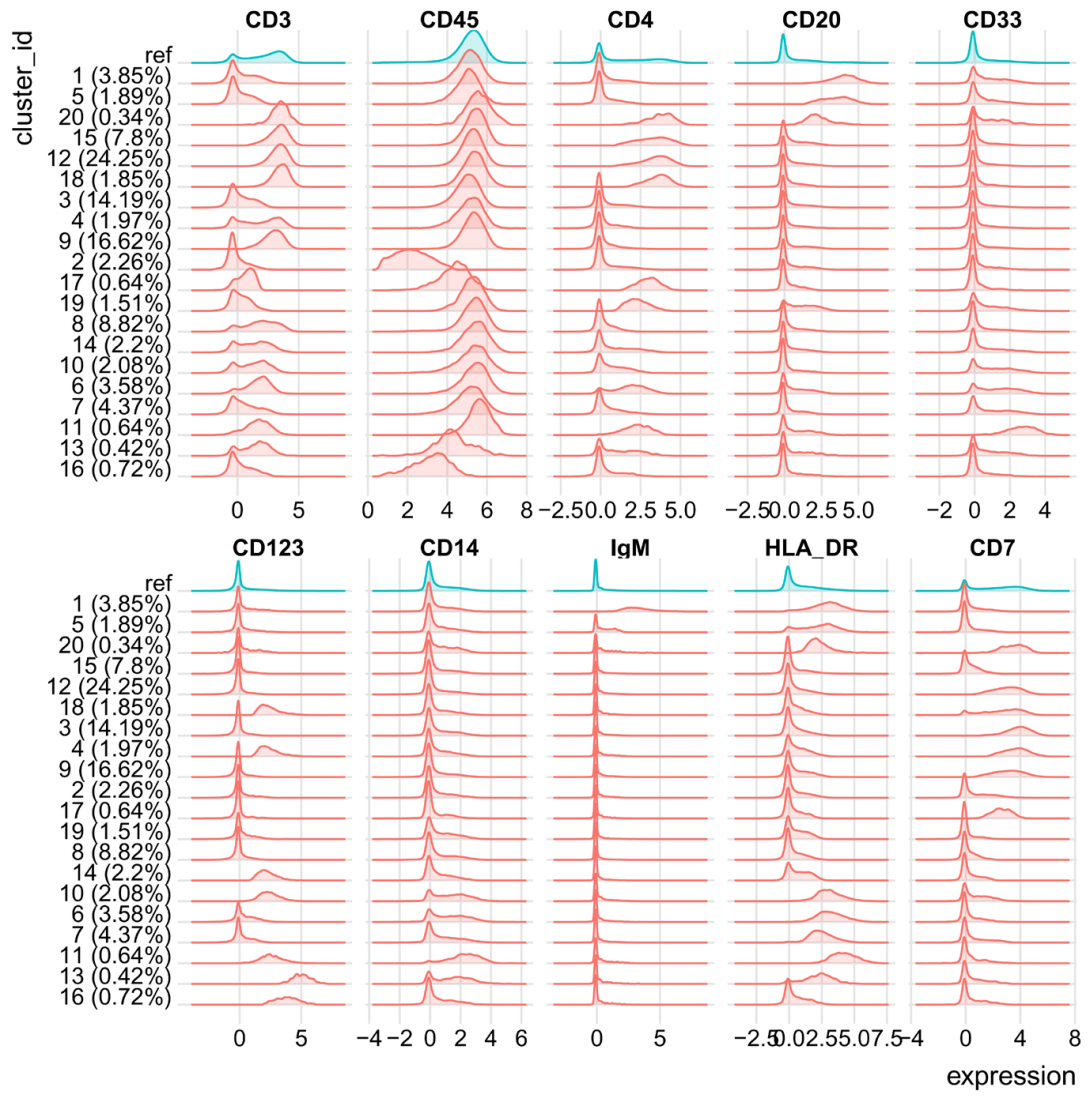
Distributions of marker intensities (arcsinh-transformed) of the 10 lineage markers in the 20 cell populations obtained with FlowSOM after the metaclustering step with ConsensusClusterPlus (PBMC data). Red densities represent marker expression for cells in a given cluster. Blue densities are calculated over all the cells and serve as a reference.

Instead of using only medians, which do not give a full representation of cluster specifics, one can plot the entire marker expression distribution in each cluster ( Figure 7). Such a plot gives more detailed profile of each cluster, but represents a larger amount of information to interpret. Heatmaps give an overall overview of clusters, are quicker and easier to interpret, and together with the dendrogram can be a good basis for further cluster merging (see Cluster merging and annotation section).

plotClusterHeatmap(daf,                               
    hm2 = NULL, k = "meta20", m = NULL,               
    cluster_anno = TRUE, draw_freqs = TRUE)           
                   
plotClusterExprs(daf, k = "meta20", markers = "type")  

**Figure 8.**
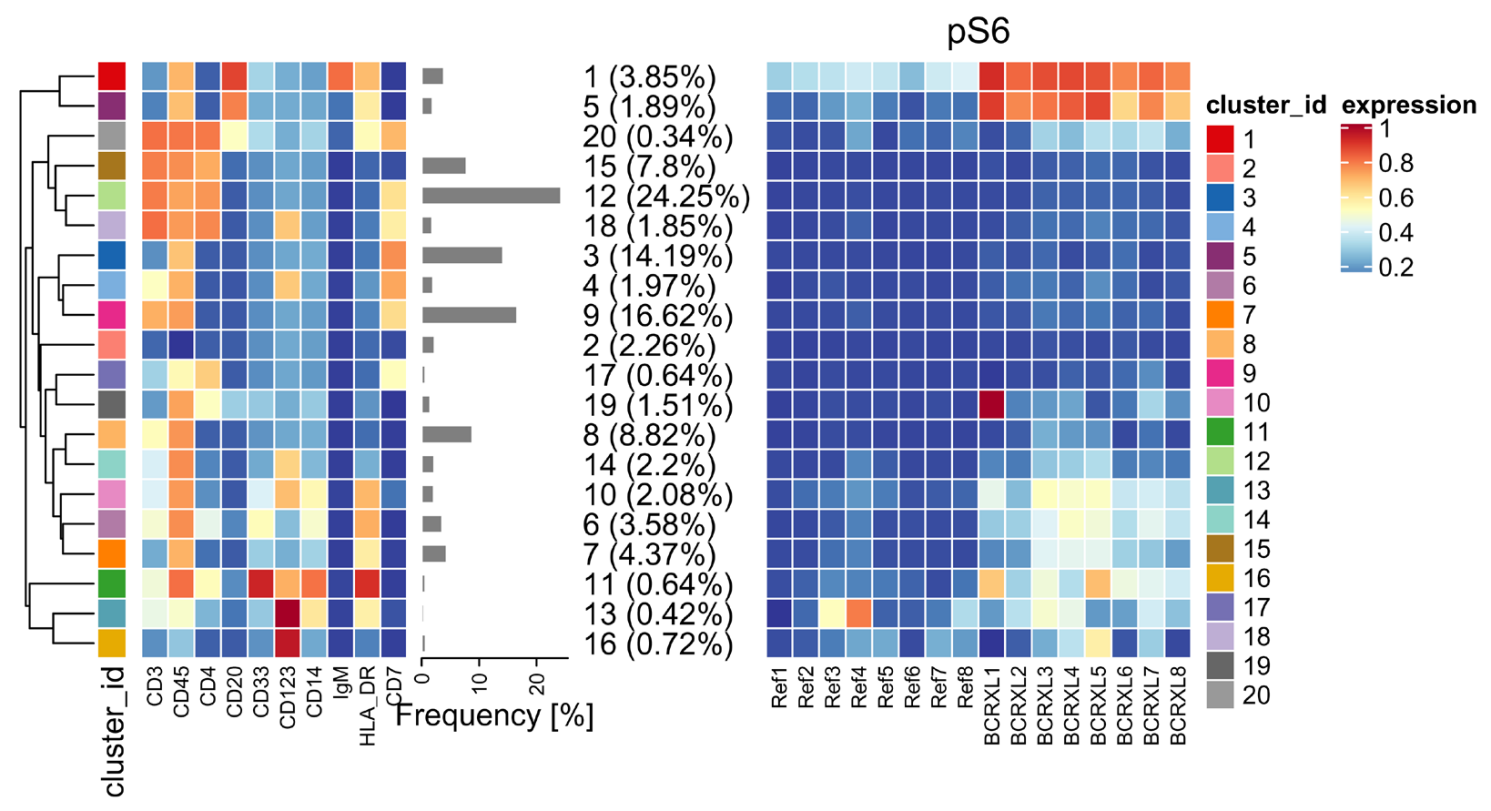
Heatmap of the median marker intensities of the 10 lineage markers and one signaling marker (pS6) across the 20 cell populations obtained with FlowSOM after the metaclustering step with ConsensusClusterPlus (PBMC data). The left panel presents a heatmap analogous to the one in Figure 6. Heatmap on the right represents the median of the arcsinh, 0-1 transformed marker expression for a signaling marker pS6 calculated over cells in each sample (columns) individually.

In addition to investigating expression of the lineage markers, we can also have a look at expression of the functional markers. We propose a heatmap that depicts median expression of functional markers in each sample ( Figure 8) such that the potential differential expression can be investigated already at this data exploration step before the formal testing is done. In order to plot all the heatmaps in one panel, we use the *ComplexHeatmap* package ^16^.

plotClusterHeatmap(daf, hm2 = "pS6", k = "meta20", draw_freqs = TRUE)

### Visual representation with UMAP

One of the most popular plots for representing single cell data are t-SNE plots, where each cell is represented in a lower, usually two-dimensional, space computed using t-stochastic neighbor embedding (t-SNE) ^34, 35^. More generally, dimensionality reduction techniques represent the similarity of points in 2 or 3 dimensions, such that similar objects in high-dimensional space are also similar in lower dimensional space. Mathematically, there are a myriad of ways to define this similarity. For example, principal component analysis (PCA) uses linear combinations of the original features to find orthogonal dimensions that show the highest levels of variability; the top 2 or 3 principal components can then be visualized.

Nevertheless, there are a few notes of caution when using t-SNE or any other dimensionality reduction technique. Since they are based on preserving similarities between cells, those that are similar in the original space will be close in the 2D/3D representation, but the opposite does not always hold. In our experience, t-SNE with default parameters for HDCyto data is often suitable (for more guidance on the specifics of t-SNE, see How to Use t-SNE Effectively ^36^).

Another nonlinear dimensionality reduction technique, uniform manifold approximation and projection (UMAP), has recently been directly compared to t-SNE, and shown to outperform t-SNE in runtime, reproducibility, and its ability to organize cells into meaningful clusters ^37, 38^. Throughout this workflow, we use UMAP as our dimensionality reduction method of choice, but other techniques, such as PCA, diffusion maps ^19^, SIMLR ^20^, isomaps or t-SNE could be applied. Alternative algorithms, such as *largeVis* ^39^ (available via the *largeVis* package) or hierarchical stochastic neighbor embedding (HSNE) ^40^, can also be used for dimensionality reduction of very large datasets without downsampling. Alternatively, the dimensionality reduction can be performed on the *codes* of the SOM, at a resolution (size of the SOM) specified by the user ( Figure 13).

*CATALYST* provides a flexible wrapper, runDR(), to perform the set of dimensionality reduction methods available in the *scater* package. To make results reproducible, the random seed should be set via set.seed *prior* to calling runDR(). The subset of markers to use for computing reduced dimensions is specified via cols_to_use, but will default to the set of type-markers defined in the input daFrame if unspecified ( type_markers(daf)); the cells to use are specified with rows_to_use. When a single numeric value N is provided, runDR() will draw a random subset of N cells per sample.

Most dimensionality reduction techniques require significant computational time to process the data. To keep running times reasonable for larger CyTOF datasets, one may use a subset of cells. The methods in *CATALYST* have been implemented in a way that allows using different sets of cells for different algorithms. For example, here we use 500 and 1000 cells from each sample to run t-SNE and UMAP, respectively.

# run t-SNE & UMAP                           
set.seed(1234)                               
daf <- runDR(daf, "TSNE", rows_to_use = 500) 
daf <- runDR(daf, "UMAP", rows_to_use = 1000)

**Figure 9.**
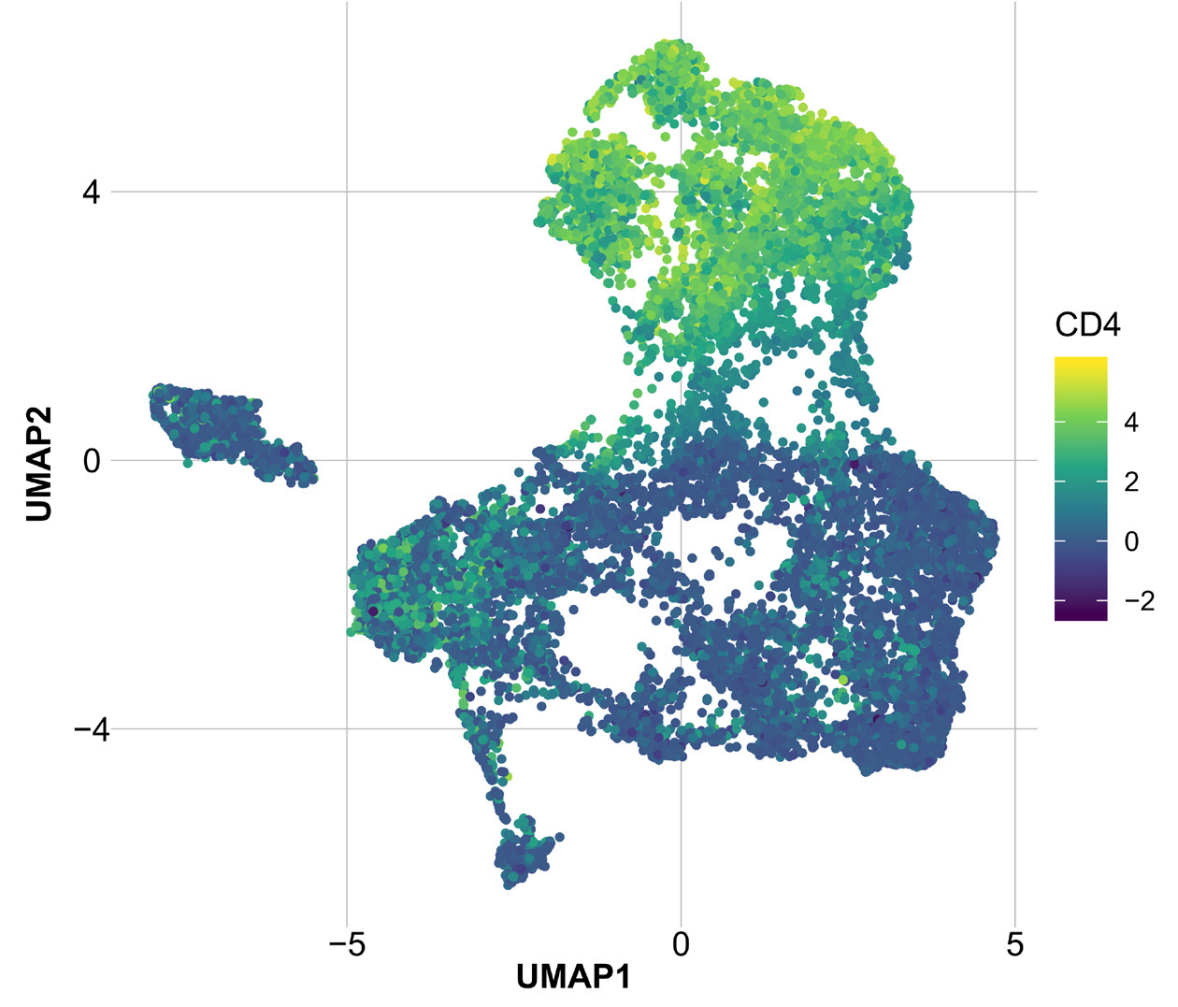
UMAP based on the arcsinh-transformed expression of the 10 lineage markers in the cells from the PBMC dataset. UMAP was run with no PCA step. From each of the 16 samples, 1000 cells were randomly selected. Cells are colored according to the expression level of the CD4 marker.

The UMAP map below is colored according to the expression level of the CD4 marker, highlighting the position of CD4+ T-cells ( Figure 9). In this way, one can use a set of markers to highlight where cell types of interest are located on the map. If one is loosely interpreting *density* of points in the map, it is recommended to select a fixed number of cells per sample.

plotDR(daf, "UMAP", color_by = "CD4")

**Figure 10.**
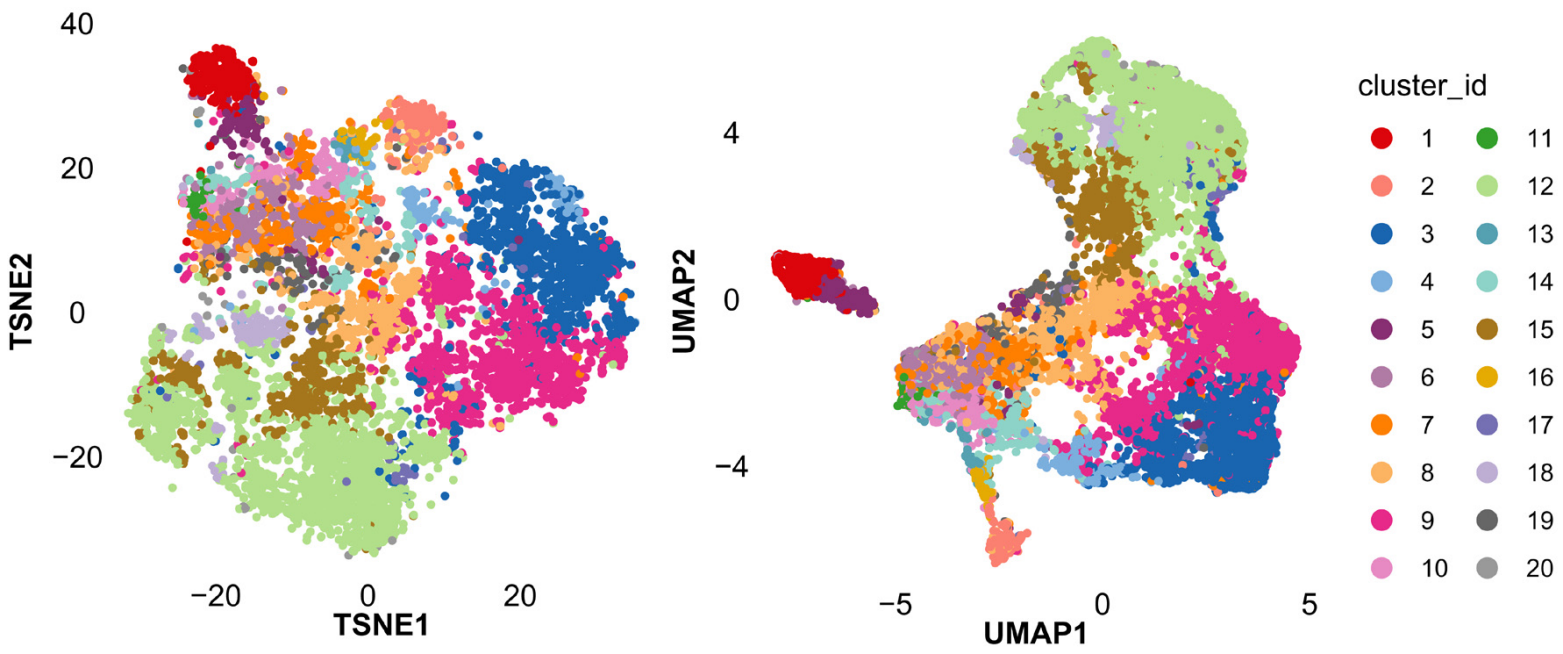
t-SNE and UMAP based on the arcsinh-transformed expression of the 10 lineage markers in the cells from the PBMC dataset. From each of the 16 samples, 500 (t-SNE) and 1000 (UMAP) cells were randomly selected. Cells are colored according to the 20 cell populations obtained with FlowSOM after the metaclustering step with ConsensusClusterPlus.

**Figure 11.**
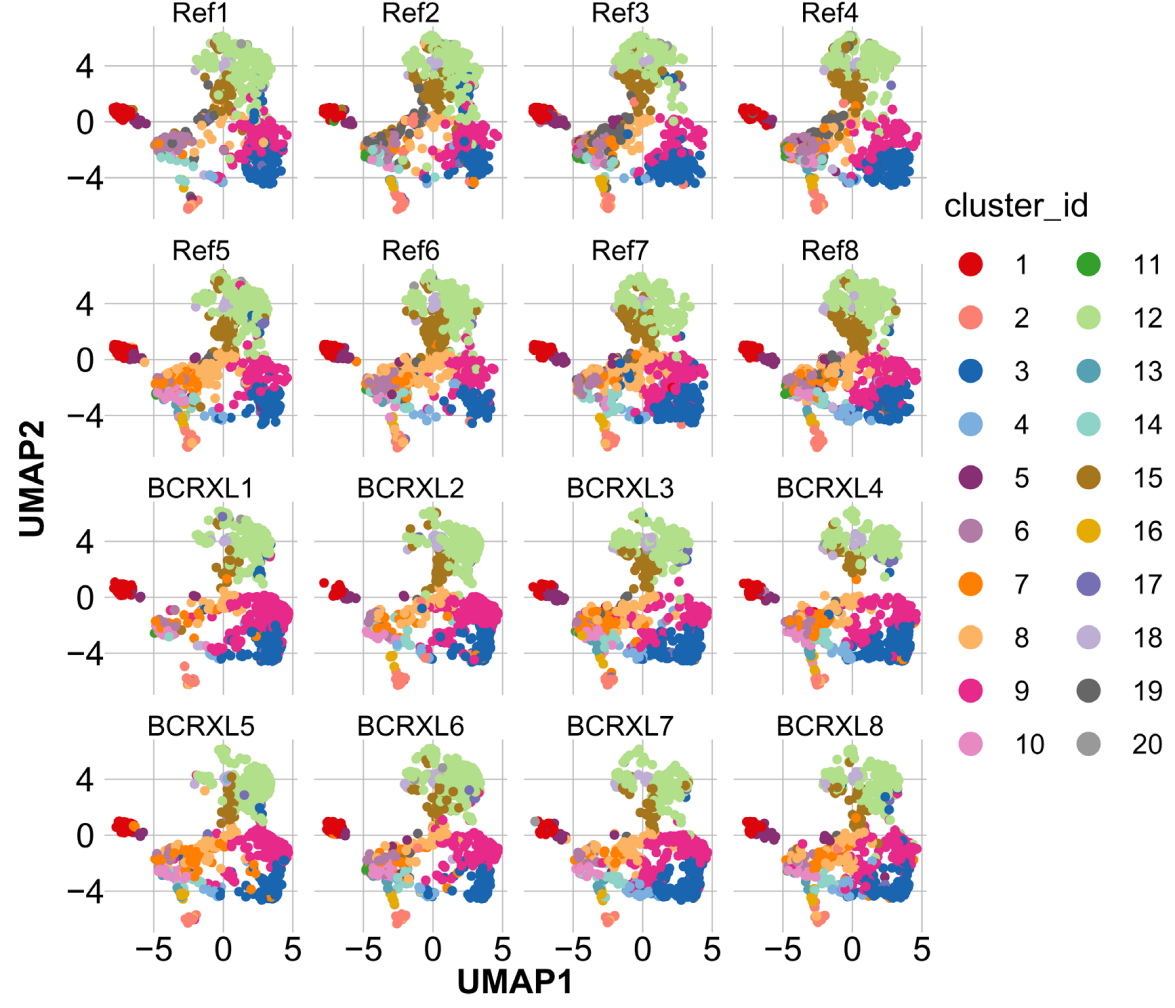
UMAP as in Figure 10, but stratified by sample.

**Figure 12.**
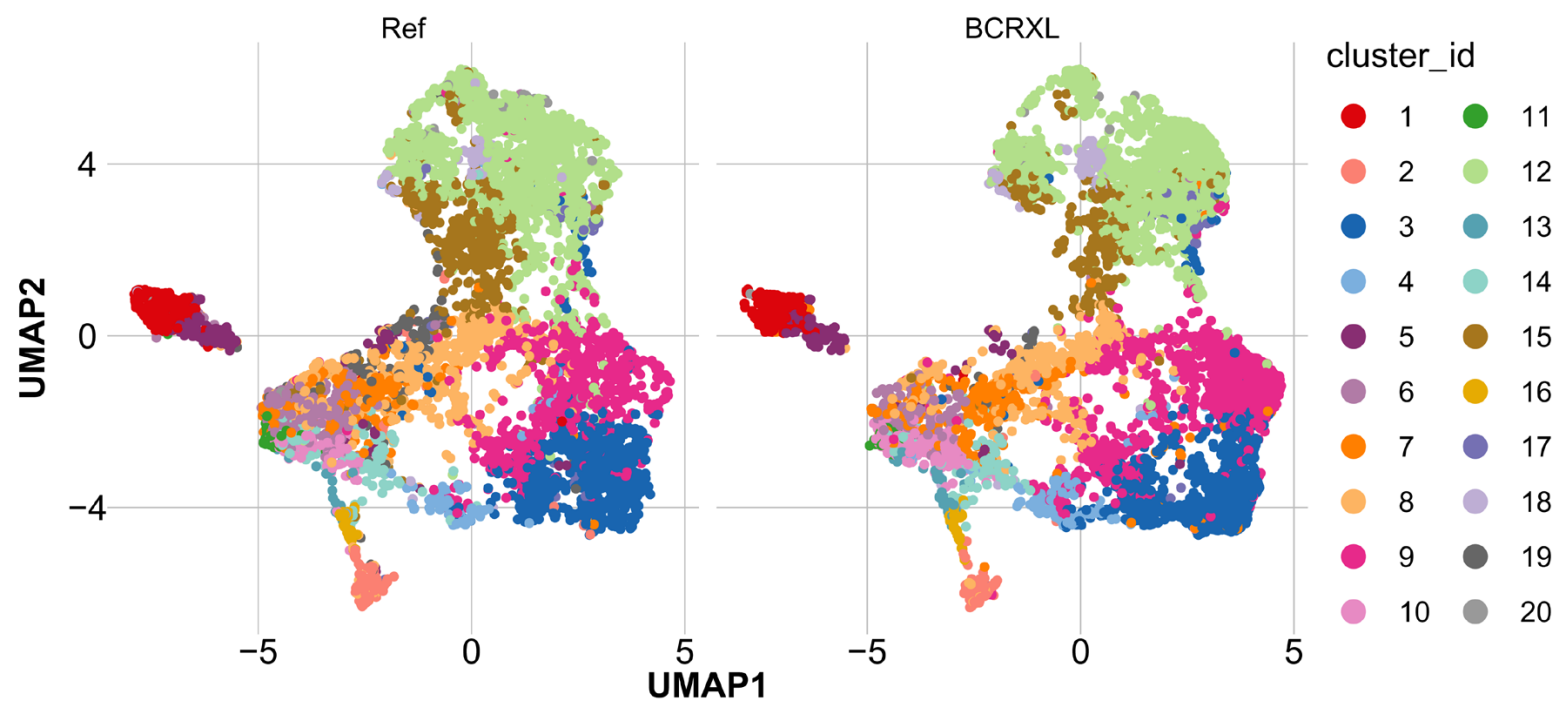
UMAP as in Figure 10, but stratified by condition.

Alternatively, we can color the cells by any resolution of clustering available in the codes. Here, we compare the t-SNE and UMAP projections of cells colored by the 20 metaclusters. Ideally, cells of the same color should be close to each other ( Figure 10). When the plots are further stratified by sample ( Figure 11), we can verify whether similar cell populations are present in all replicates, which can help in identifying outlying samples. Optionally, stratification can be done by condition ( Figure 12). With such a spot-check plot, we can inspect whether differences in cell abundance are strong between conditions, and we can visualize and identify distinguishing clusters before applying formal statistical testing. A similar approach of data exploration was proposed in studies of treatment-specific differences of polyfunctional antigen-specific T-cells ^41^.

p1 <- plotDR(daf, "TSNE", color_by = "meta20") +             
    theme(legend.position = "none")                          
p2 <- plotDR(daf, "UMAP", color_by = "meta20")               
lgd <- get_legend(p2)                                        
p2 <- p2 + theme(legend.position = "none")                   
plot_grid(p1, p2, lgd, nrow = 1, rel_widths = c(5, 5, 2))    

## Facet per sample                                          
plotDR(daf, "UMAP", color_by = "meta20", facet = "sample_id")

## Facet per condition                                       
plotDR(daf, "UMAP", color_by = "meta20", facet = "condition")

**Figure 13.**
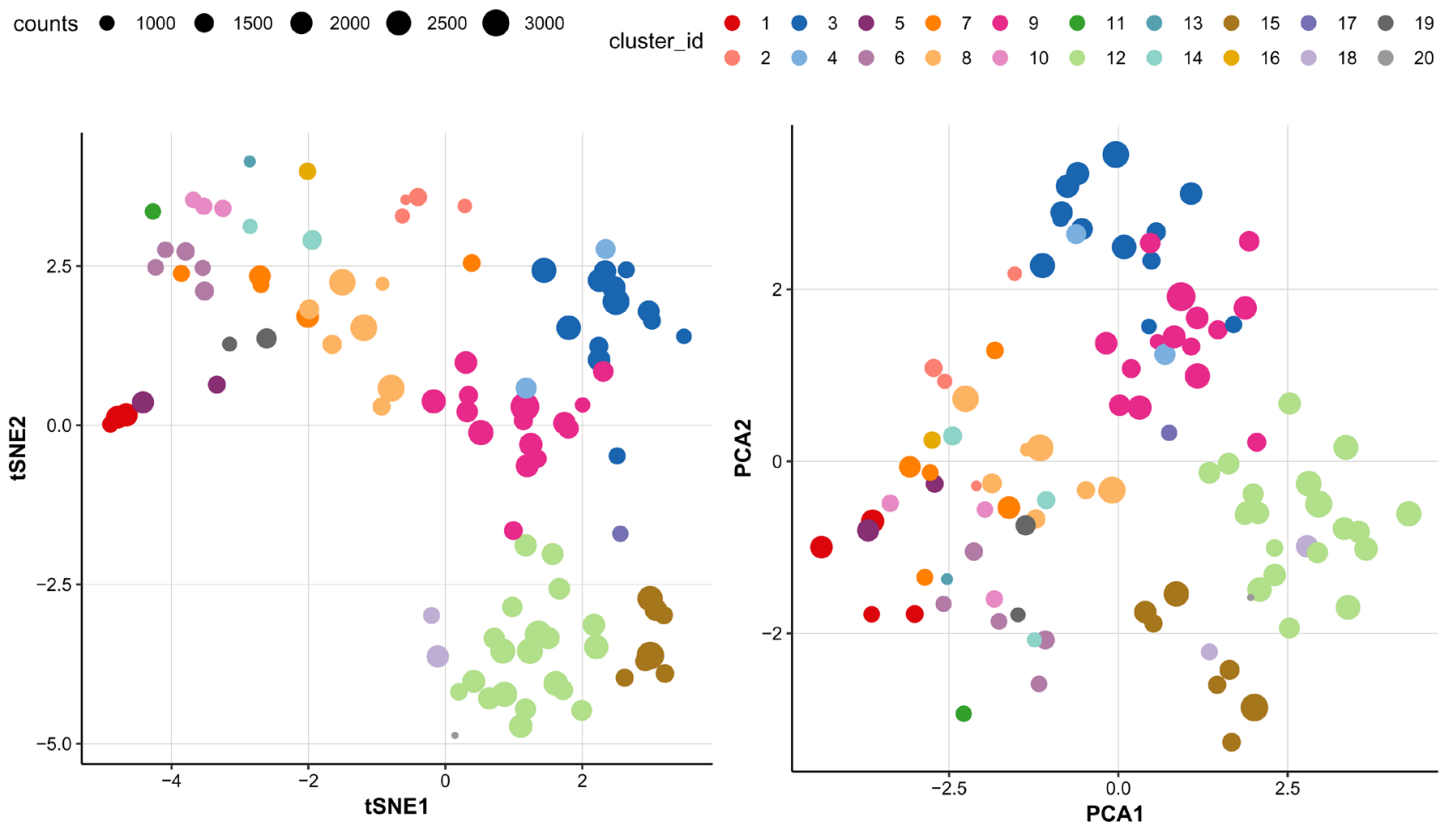
The 100 SOM codes in the PBMC dataset colored according to the metaclustering with Consen-susClusterPlus into 20 cell populations presented after the dimension reduction with ( **A**) t-SNE and ( **B**) PCA. The SOM codes represent characteristics of the 100 (by default) clusters generated in the first step of the FlowSOM pipeline. The size of the points corresponds to the number of cells that were assigned to a given code.

The SOM codes represent characteristics of the 100 (by default) clusters generated in the first step of the *FlowSOM* pipeline. Their visualization can also be helpful in understanding the cell population structure and determining the number of clusters. Ultimately, the metaclustering step uses the codes and not the original cells. We treat the codes as new representative cells and apply the t-SNE dimension reduction to visualize them in 2D ( Figure 13). The size of the points corresponds to the number of cells that were assigned to a given code. The points are colored according to the results of metaclustering. Since we have only 100 data points, the t-SNE analysis is fast.

As there are multiple ways to mathematically define similarity in high-dimensional space, it is always good practice visualizing projections from other methods to see how consistent the observed patterns are. For instance, we also represent the *FlowSOM* codes via the first two principal components ( Figure 13).

plotCodes(daf, k = "meta20")

**Figure 14.**
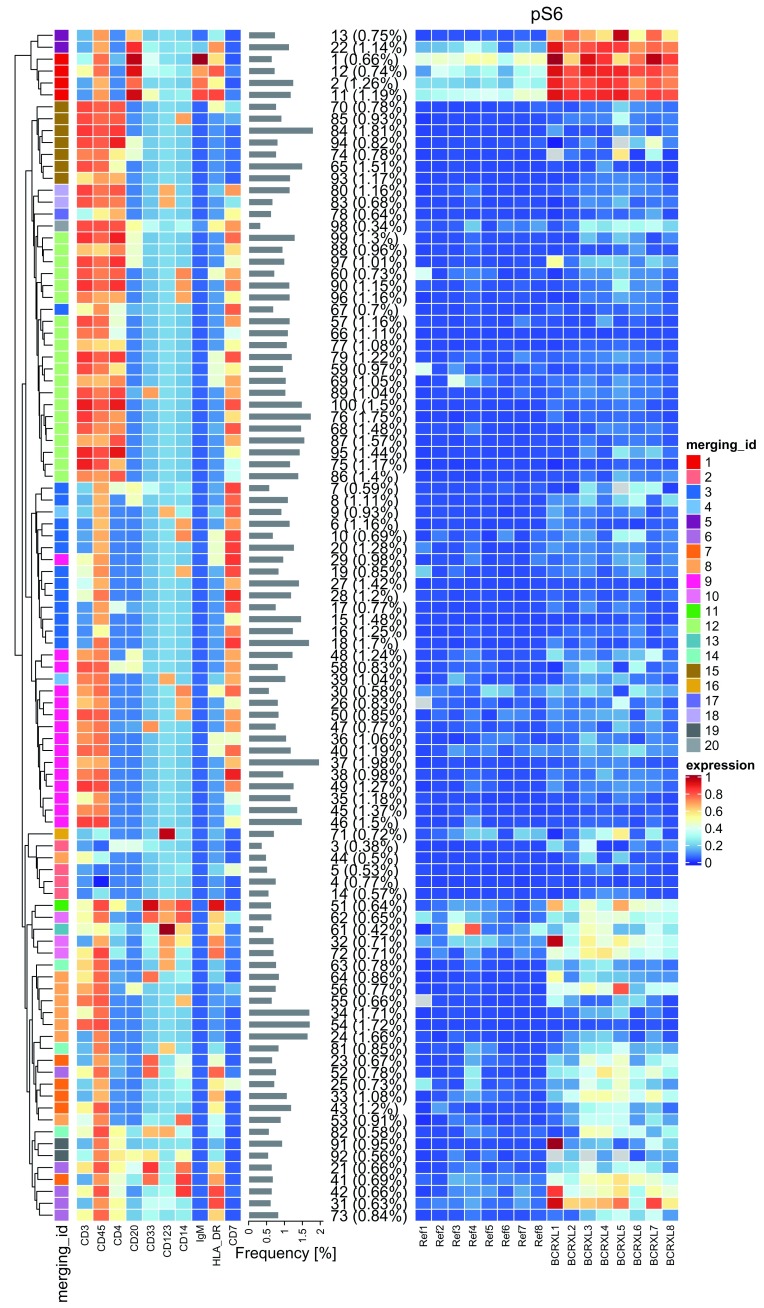
Heatmap of the median marker intensities of the 10 lineage markers (left panel) and one signaling marker pS6 (right panel) across the 100 SOM codes in the PBMC dataset. The color in the heatmap represents The color in the heatmap represents the median of the arcsinh, 0-1 transformed marker expression calculated over cells from all the samples, for the lineage markers, and over cells in each sample individually, for the signaling marker. The heat varies from blue for lower expression to red for higher expression. The dendrogram on the left represents the hierarchical similarity between the 100 codes (metric: Euclidean distance; linkage: average). The annotation bar on the left is colored according to the code metaclustering with ConsensusClusterPlus into 20 cell populations. The relative size of the codes is shown in parentheses next to the cluster numbers.

Using heatmaps, we can also visualize median marker expression in the 100 SOM codes as in Figure 14. Of note, the clustering presented with the dendrogram does not completely agree with the clustering depicted by the 20 colors because the first one is based on the hierarchical clustering with average linkage and Euclidean distance, while the second one results from the consensus clustering.

plotClusterHeatmap(daf,                     
    hm2 = "pS6", k = "som100", m = "meta20",
    cluster_anno = FALSE, draw_freqs = TRUE)

**Figure 15.**
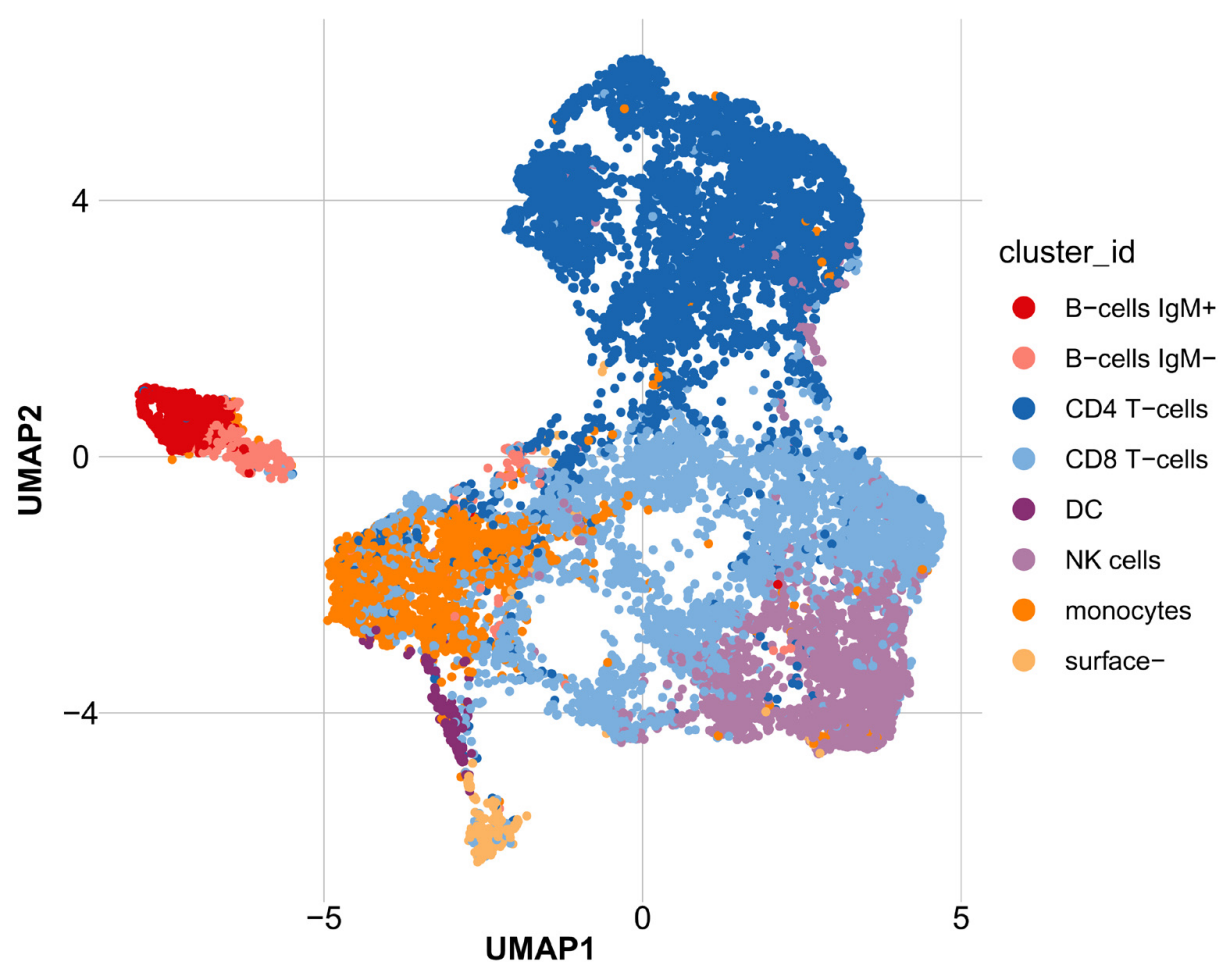
UMAP plot for the PBMC dataset, where cells are colored according to the manual merging of the 20 cell populations, obtained with FlowSOM, into 8 PBMC populations. As in 10, UMAP uses the arcsinh-transformed expression of the 10 lineage markers in 1000 randomly selected cells from each of the 16 samples.

### Cluster merging and annotation

In our experience, manual merging of clusters leads to slightly different results compared to an algorithm with a specified number of clusters. In order to detect somewhat rare populations, some level of over-clustering is necessary so that the more subtle populations become separated from the main populations. In addition, merging can always follow an over-clustering step, but splitting of existing clusters is not generally feasible.

**Figure 16.**
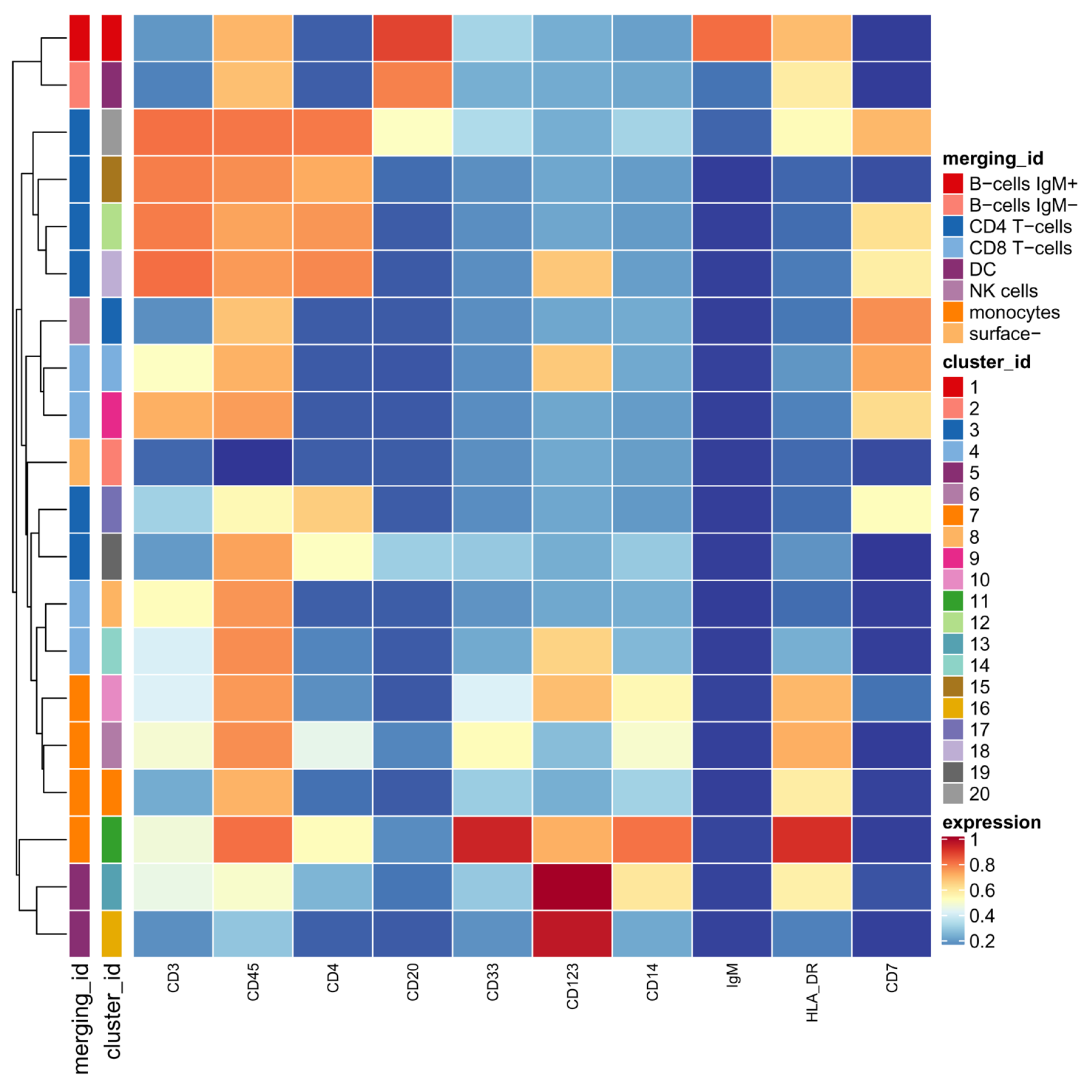
Heatmap as in Figure 6, where the additional color bar on the left indicates how the 20 metaclusters, obtained with FlowSOM, are merged into the 8 PBMC populations.

In our setup, over-clustering is also useful when the interest is identifying the “natural” number of clusters present in the data. In addition to heatmaps and UMAP plots, one could investigate the delta area plot from the *ConsensusClusterPlus* package and the hierarchical clustering dendrogram of the over-clustered subpopulations, as shown in Figure 16 and Figure 18.

In our example, we expect around 6 specific cell types, and we have performed *FlowSOM* clustering into 20 groups as a reasonable over-estimate. After analyzing the heatmaps ( Figure 6) and UMAP plots ( Figure 10), we can clearly see that stratification of the data into 20 clusters may be too strong. In the UMAP plot, many clusters are placed very close to each other, indicating that they could be merged together. The same can be deduced from the heatmaps, highlighting that marker expression patterns for some neighboring clusters are very similar. Cluster merging and annotation is somewhat manual, based partially on visual inspection of UMAP plots and heatmaps and thus, benefits from expert knowledge of the cell types.

***Manual cluster merging and annotation based on heatmaps.*** Our main reference for manual merging of clusters is the heatmap of marker characteristics across metaclusters (e.g., Figure 6), with dendrograms showing the hierarchy of similarities. Such plots show cluster- or cluster-sample level information, and thus aggregate marker expression across many cells. Together with dimensionality reduction, these plots give good insight into the relationships between clusters and the marker levels within each cluster. Given expert knowledge of the cell types and markers, it is then left to the researcher to decide how exactly to merge clusters (e.g., with higher weight given to some markers). The dendrogram highlights the similarity between the metaclusters and can be used explicitly for the merging. However, there are reasons why we would not always follow the dendrogram exactly. In general, when it comes to clustering, blindly following the hierarchy of codes will lead to identification of populations of similar cells, but it does not necessarily mean that they are of biological interest. The distances between metaclusters are calculated across all the markers, and it may be that some markers carry higher weight for certain cell types. In addition, different linkage methods may lead to a different hierarchy, especially when clusters are not fully distinct. Another aspect to consider in cluster merging is the cluster size, represented in the parentheses next to the cluster label in our plots. If the cluster size is very small, but the cluster seems relevant and distinct, one can keep it as separate. However, if it is small and different from the neighboring clustering only in a somewhat unimportant marker, it could be merged. And, if some of the metaclusters do not represent any specific cell types, they could be dropped out of the downstream analysis instead of being merged. However, in case an automated solution for cluster merging is truly needed, one could use the cutree() function applied to the dendrogram.

Based on the set random seed, a manual merging of the 20 metaclusters is defined in PBMC8_cluster_merging1.xlsx available at the Robinson Lab server. This merging table contains, for each of the original clusters, an ID to newly assign to cells assigned to the given cluster. Clusters may be merged with the mergeClusters() function from *CATALYST*. For future reference, each manual merging is assigned an ID specified with argument id. Note that, if multiple old clusters are given the same new label, the respective clusters will be merged.

In this example, our expert has annotated 8 cell populations: CD8 T-cells, CD4 T-cells, B-cells IgM-, B-cells IgM+, NK cells, dendritic cells (DCs), monocytes and surface negative cells; monocytes could be further subdivided based on HLA-DR into high, medium and low subtypes.

merging_table1 <- "PBMC8_cluster_merging1.xlsx"       
download.file(file.path(url, merging_table1),         
    destfile = merging_table1, mode = "wb")           
merging_table1 <- read_excel(merging_table1)          
head(data.frame(merging_table1))                      

##   original_cluster  new_cluster
## 1                1 B-cells IgM+
## 2                2     surface-
## 3                3     NK cells
## 4                4  CD8 T-cells
## 5                5 B-cells IgM-
## 6                6    monocytes

# convert to factor with merged clusters in desired order                
merging_table1$new_cluster <- factor(merging_table1$new_cluster,         
    levels = c("B-cells IgM+", "B-cells IgM-", "CD4 T-cells",             
       "CD8 T-cells", "DC", "NK cells", "monocytes", "surface-"))        
                                                                         
daf <- mergeClusters(daf, k = "meta20",                                  
    table = merging_table1, id = "merging1")                             

We can view the UMAP plot with the new annotated cell populations by specifying color_by = "merging1" ( Figure 15).

plotDR(daf, "UMAP", color_by = "merging1")

One of the useful representations of merging is a heatmap of median marker expression in each of the original clusters, which are labeled according to the proposed merging, Figure 16. As before, the clustering to use for computing cluster medians is specified with k = "meta20". For visualization, we can specify a second layer of cluster annotations with m = "merging1".

plotClusterHeatmap(daf, k = "meta20", m = "merging1")

**Figure 17.**
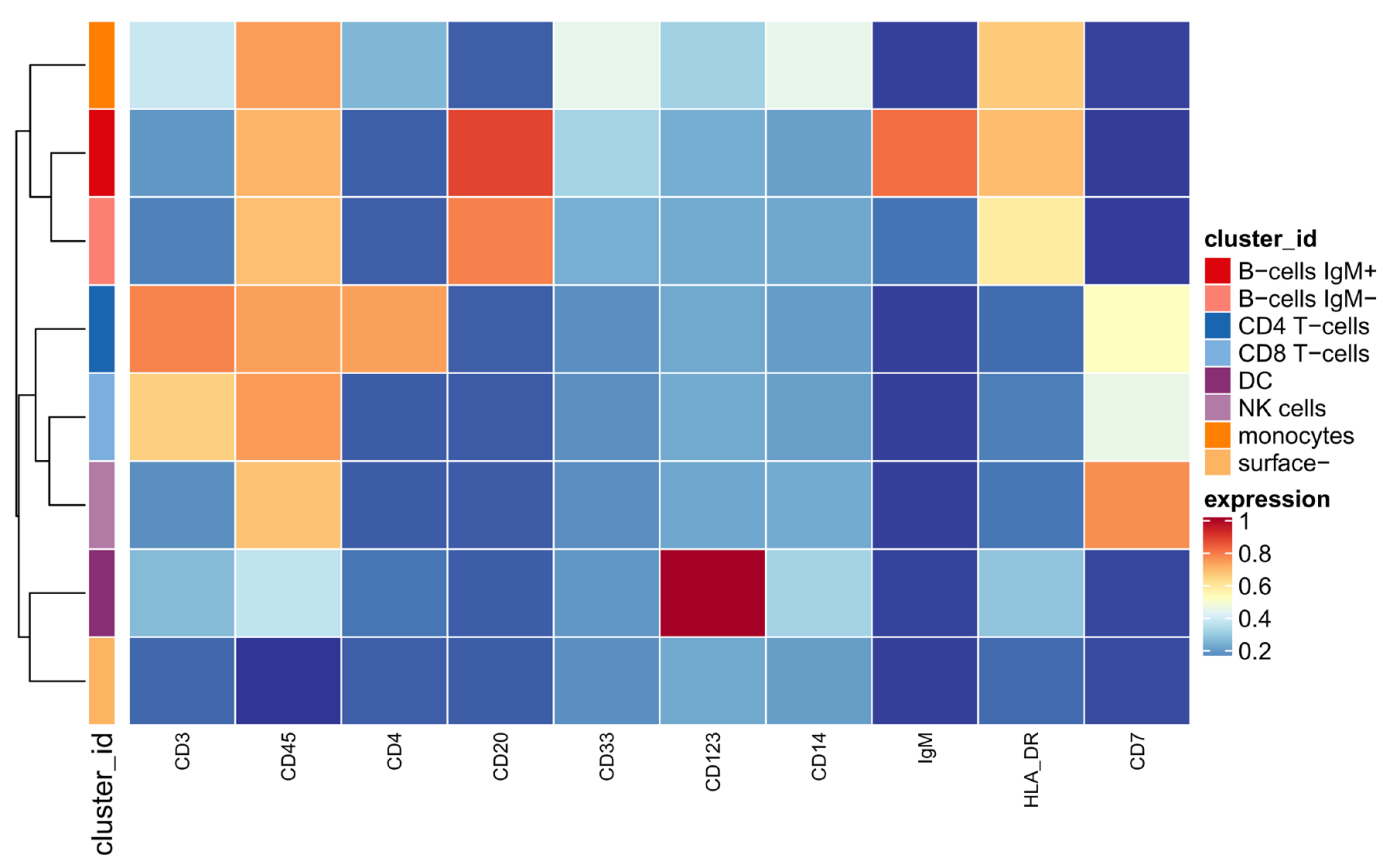
Heatmap of the median marker intensities of the 10 lineage markers in the 8 PBMC cell populations obtained by manual merging of the 20 metaclusters generated by FlowSOM. As in Figure 6, the heat represents the median of arcsinh and 0-1 transformed marker expression calculated over cells from all the samples. The dendrogram on the left represents the hierarchical similarity between the 8 populations calculated using Euclidean distance and average linkage.

To get a final summary of the annotated cell types, we can plot a heatmap of median marker expressions that are calculated based on the manual merging’s cluster annotations ( Figure 17).

plotClusterHeatmap(daf, k = "merging1")

**Figure 18.**
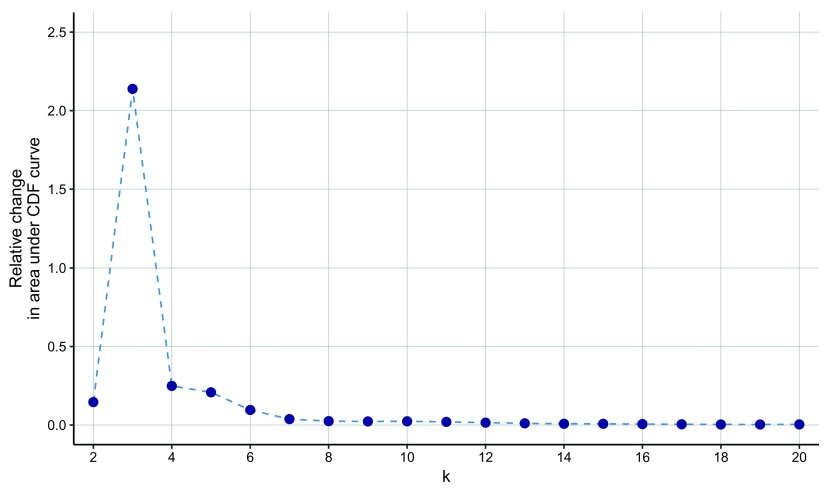
The delta area plot generated in the metaclustering step by the ConsensusClusterPlus function. The delta area score (y-axis) indicates the relative increase in cluster stability obtained when clustering the 100 SOM codes generated by FlowSOM into k groups (x-axis).

***Reducing the number of clusters in ConsensusClusterPlus.*** The *ConsensusClusterPlus* package provides visualizations that can help to understand the metaclustering process and the characteristics of the analyzed data. For example, the delta area plot ( Figure 18) highlights the amount of extra cluster stability gained when clustering into k groups as compared to k-1 groups (from k=2 to k=20). It can be expected that high stability of clusters can be reached when clustering into the number of groups that best fits the data. Thus, using the delta area plot could help finding the “natural” number of clusters present in the data, which would correspond to the value of k where there is no appreciable increase in stability. This strategy can be referred as the “elbow criterion”. For more details about the meaning of this plot, the user can refer to the original description of the consensus clustering method ^42^.

The elbow criterion is quite subjective since the “appreciable” increase is not defined exactly. For example, in the delta plot below, we could say that the last point before plateau is for k=6, or for k=5, or for k=3, depending on our perception of sufficient decrease of the delta area score. Moreover, the exact point where a plateau is reached may vary for runs with different random seeds, the drop may not always be so sharp and the function is not guaranteed to be decreasing. It is advisable to investigate more of these plots and the resulting UMAP and heatmaps before drawing any conclusions about the final number of “natural” clusters.

Manual merging of up to 20 clusters can be laborious. To simplify this task, one could reduce the strength of over-clustering and allow the metaclustering method to do a part of the merging, which then can be completed manually. Analyzing the delta plot from the right side, we can see how much we can reduce the strength of over-clustering while still obtaining stable clusters. In parallel, one should check the heatmaps to see whether the less stringent stratification is able to capture cell populations of interest.

As an example, we consider the metaclustering to 12 groups. Clustering into as few as 12 groups still allows us to identify the same 8 cell populations as when merging 20 clusters, but these are simpler to define since fewer profiles need to be manually scanned. The expert-based merging of the 12 metaclusters into 8 PBMC cell populations is saved in the PBMC8_cluster_merging2_v3.xlsx file on the Robinson Lab server.

merging_table2 <- "PBMC8_cluster_merging2_v3.xlsx"     
download.file(file.path(url, merging_table2),          
    destfile = merging_table2, mode = "wb")            
merging_table2 <- read_excel(merging_table2)           
data.frame(merging_table2)                             

##   original_cluster   new_cluster
## 1                1  B-cells IgM+
## 2                2      surface-
## 3                3      NK cells
## 4                4   CD8 T-cells
## 5                5  B-cells IgM-
## 6                6     monocytes
## 7                7   CD8 T-cells
## 8                8     monocytes
## 9                9   CD4 T-cells
## 10              10            DC
## 11              11   CD4 T-cells
## 12              12   CD4 T-cells

# convert to factor with merged clusters in desired order       
merging_table2$new_cluster <- factor(                           
    merging_table2$new_cluster,                                 
    levels = levels(merging_table1$new_cluster))                
                                                                
# apply 2nd manual merging                                      
daf <- mergeClusters(daf, k = "meta12",                         
    table = merging_table2, id = "merging2")                    

***Comparison of automated and manual merging.*** The manual merging of 20 (or 12) clusters by an expert resulted in identification of 8 cell populations. To highlight the impact of manual merging versus algorithm-defined subpopulations, we compare to the results of an automated cluster merging that is set to stratify the data also into 8 clusters. Out of interest, we can see which clusters are split by tabulating the cell labels.

# tabular comparison of algorithmic & manual merging             
table(manual = cluster_codes(daf)[cluster_ids(daf), "merging2"], 
    algorithm = cluster_codes(daf)[cluster_ids(daf), "meta8"] )  

##               algorithm
## manual             1     2     3     4     5     6    7    8
##   B-cells IgM+  6651     0     0     0     0     0    0    0
##   B-cells IgM-  3265     0     0     0     0     0    0    0
##   CD4 T-cells      0     0  1203     0  2603 59174    0 1113
##   CD8 T-cells      0     0 32112     0 19038     0    0    0
##   DC               0     0     0     0     0     0 1980    0
##   NK cells         0     0 23315     0     0     0    0    0
##   monocytes        0     0     0 18436     0     0    0    0
##   surface-         0  3901     0     0     0     0    0    0

**Figure 19.**
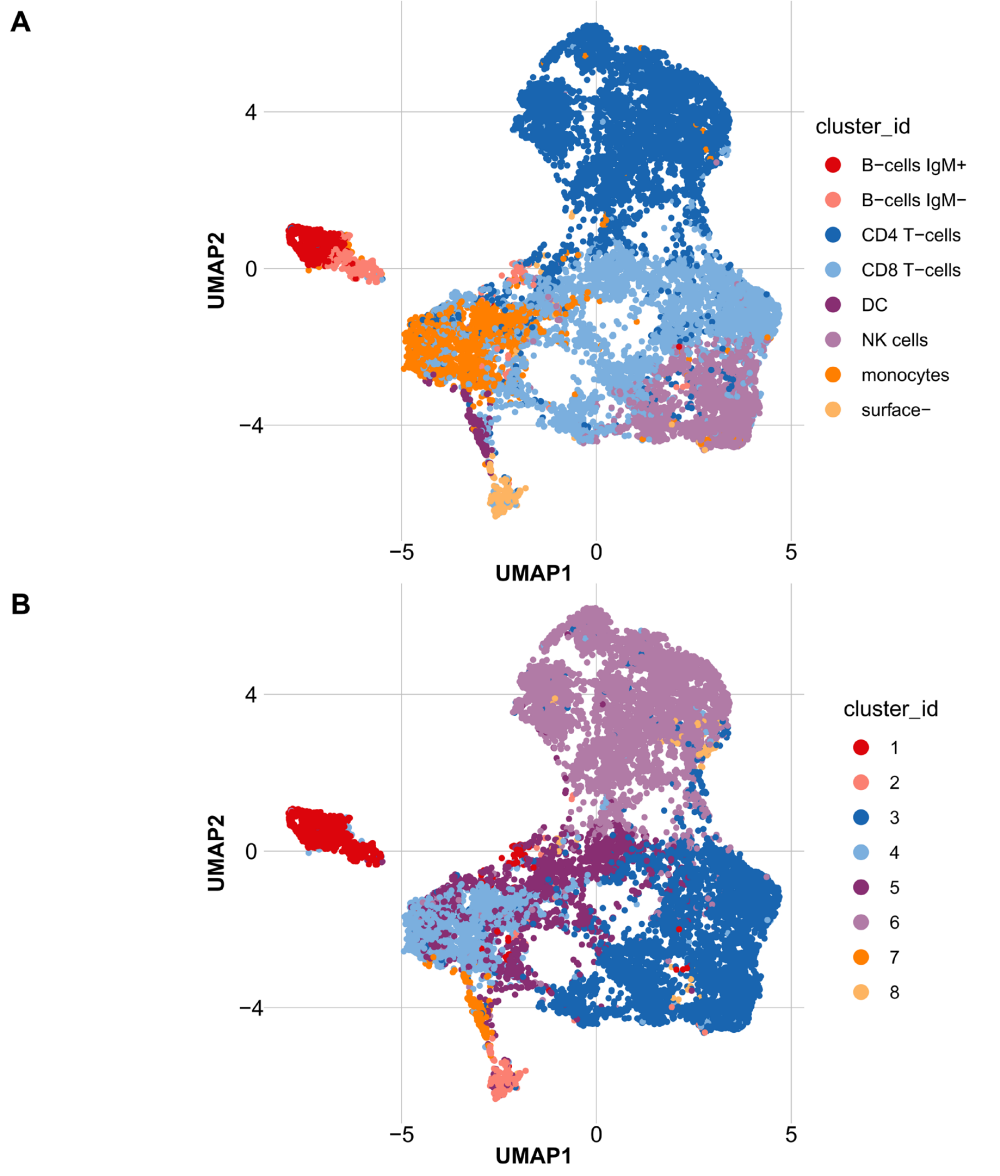
UMAP plot with cells colored according to ( **A**) the expert merging of 12 metaclusters obtained with FlowSOM into 8 PBMC populations; and ( **B**) the 8 automatically detected with FlowSOM metaclusters.

In the UMAP plot ( Figure 19), we can see that part of the new cell populations (cluster 6, 1, 2, 4 and 7) overlap substantially with populations obtained by the means of manual merging (CD4 T-cells, B-cells, surface-, monocytes and DC). However, cells that belong to clusters 3 and 5 are subdivided in a different manner according to the manual merging. Cluster 1 consists of both B-cells IgM+ and IgM- and is not further subdivided, whereas cluster 8 is altogether unidentifiable.

p1 <- plotDR(daf, "UMAP", color_by = "merging2")              
p2 <- plotDR(daf, "UMAP", color_by = "meta8")                 
plot_grid(p1, p2, ncol = 1, align = "v", labels = c("A", "B"))

The brief example above highlights the difference between automatic clustering and manual merging of algorithm-generated clusters in the search for biologically meaningful cell populations. Automated and manual merging may give different weight to marker importance and thus result in different populations being detected. However, in our view, the manual merging done here in a reproducible fashion results in a more biologically meaningful cell stratification.

## Differential analysis

For the dataset used in this workflow ^8, 21^, we perform three types of analyses that aim to identify subsets of PBMCs and signaling markers that respond to BCR/FcR-XL stimulation, by comparing stimulated samples to unstimulated samples. We first describe differential abundance of the defined cell populations, followed by differential analysis of marker expression within each cluster. Finally, differential analysis of the overall aggregated marker expression could also be of interest.

The PBMC subsets analyzed in this workflow originate from a paired experiment, where samples from 8 patients were treated with 12 different stimulation conditions for 30 minutes, together with unstimulated reference samples ^21^. This is a natural example where one would choose a mixed model to model the response (abundance or marker signal), and patients would be treated as a random effect. In this way, one can formally account for between-patient variability, observed to be quite strong in the MDS plot ( MDS plot section), and this should give a gain in power to detect differences between conditions.

We use the *diffcyt* package ^13^ to perform the differential analyses. This package includes implementations of various methods for differential testing, including linear mixed models. The mixed models methodology uses the *stats* and *lme4* packages to fit the fixed and mixed models, respectively, and the *multcomp* package for hypothesis testing.

In all differential analyses here, we want to test for differences between the reference ( Ref) and BCR/FcR-XL treatment ( BCRXL). The fixed model formula is straightforward: ~ condition, where condition indicates the treatment group. The corresponding full model design matrix consists of the intercept and dummy variable indicating the treated samples. In the presence of covariates (e.g., batch), one can include them in the model by using a formula ~ condition + covariate, or if they affect the treatment, a formula with interactions ~ condition * covariate. When using the *diffcyt* package, the model formula can be set up in the required format using the createFormula() function.

For testing, the mixed models methodology uses the general linear hypotheses function glht() from the *multcomp* package, which allows testing of arbitrary hypotheses using t-tests. In our analysis, the contrast indicates a regression coefficient to be tested equal to zero; i.e., that there is no effect of the BCR/FcR-XL treatment. The result of the test is a p-value that indicates the probability of observing an as strong (or stronger) difference between the two conditions assuming the null hypothesis is true. The linear hypotheses to be tested are specified using a contrast matrix, where the number of rows of the contrast matrix equals the number of columns of the design matrix. When using the *diffcyt* package, the contrast matrix can be created in the required format using the createContrast() function.

Testing is performed on each cluster and marker separately, resulting in 8 tests for differential abundance (one for each merged population), 14 tests for overall differential marker expression analysis and 8 x 14 tests for differential marker expression within populations. Thus, to account for the multiple testing correction, we apply the Benjamini-Hochberg adjustment to each of them using a false discovery rate (FDR) cutoff of 5%.

FDR_cutoff <- 0.05

### Differential cell population abundance

Differential analysis of cell population abundance compares the proportions of cell types across experimental conditions and aims to highlight populations that are present at different ratios. First, we calculate two tables: one that contains cell counts for each sample and population and one with the corresponding proportions of cell types by sample. The proportions are used only for plotting, since the statistical modeling takes the cell counts by cluster and sample as input.

**Figure 20.**
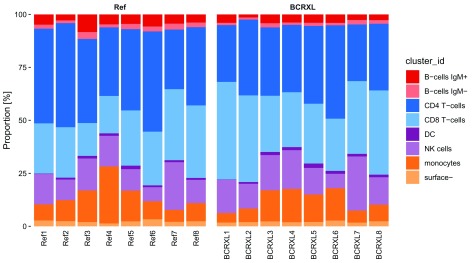
Relative abundance of the 8 PBMC populations in each sample (x-axis), in the PBMC dataset, represented with a barplot. The 8 cell populations are a result of manual merging of the 20 FlowSOM metaclusters.

For each sample, we plot its PBMC cell type composition represented with colored bars, where the size of a given stripe reflects the proportion of the corresponding cell type in a given sample ( Figure 20).

plotAbundances(daf, k = "merging1", by = "sample_id")

**Figure 21.**
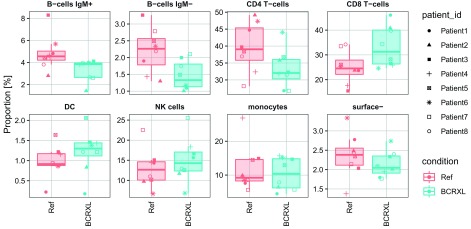
Relative abundance of the 8 PBMC populations in each sample, in the PBMC dataset, represented with boxplots. Values for the two conditions are indicated with different colors: red for the unstimulated (Ref) and blue for the stimulated with BCR/FcR-XL (BCRXL) samples. Values for each patient are indicated with different shape. The 8 cell populations are a result of manual merging of the 20 FlowSOM metaclusters.

It may be quite hard to see the differences in cluster abundances in the plot above, especially for clusters with very low frequency. And, since boxplots cannot represent multimodal distributions, we show boxplots with jittered points of the sample-level cluster proportions overlaid ( Figure 21). The y-axes are scaled to the range of data plotted for each cluster, to better visualize the differences in frequency of lower abundance clusters. For this experiment, it may be interesting to additionally depict the patient information. We do this by plotting a different point shape for each patient. Indeed, we can see that often the direction of abundance changes between the two conditions are concordant among the patients.

plotAbundances(daf, k = "merging1", by = "cluster_id", shape = "patient_id")

Since our goal is to compare proportions, one could take these values, transform them (e.g., logit) and use them as a dependent variable in a linear model. However, this approach does not take into account the uncertainty of proportion estimates, which is higher when ratios are calculated for samples with lower total cell counts. A distribution that naturally accounts for such uncertainty is the binomial distribution (i.e., logistic regression), which takes the cell counts as input (relative to the total for each sample). Nevertheless, as in genomic data analysis, pure logistic regression is not able to capture the overdispersion that is present in HDCyto data. A natural extension to model the extra variation is the generalized linear mixed model (GLMM), where the random effect is defined by the sample ID (observation-level random effects ^43, 44^). Additionally, in our example the patient pairing could be accounted in the model by incorporating a random intercept for each patient. Thus, we present two GLMMs. Both of them comprise a random effect defined by the sample ID to model the overdispersion in proportions. The second model includes a random effect defined by the patient ID to account for the experiment pairing.

In our model, the blocking variable is patient ID *i* = 1, ..., *n*, where *n* = 8. For each patient, there are *n _i_* samples measured, and *j* = 1, ..., *n _i_* indicates the sample ID. Here, *n _i_* = 2 for all *i* (one from reference and one from BCR/FcR-XL stimulated).

We assume that for a given cell population, the cell counts *Y _ij_* follow a binomial distribution *Y _ij_ ∼ Bin*( *m _ij_*, *π _ij_*), where *m _ij_* is a total number of cells in a sample corresponding to patient *i* and condition *j*.

The GLMM with observation-level random effects *ξ _ij_* is defined as follows:

$$E(Y_{ij}|\beta_{0},\beta_{1},\xi_{ij})=logit^{-1}(\beta_{0}+\beta_{1}x_{ij}+\xi_{ij}),$$

where $\xi_{ij}∼N(0,\sigma_{\xi }^{2})$ and *x _ij_* corresponds to the conditionBCRXL column in the design matrix and indicates whether a sample *ij* belongs to the reference ( *x _ij_* = 0) or treated condition ( *x _ij_* = 1). Since *E*( *Y _ij_|β*_0_, *β*_1_, *ξ _ij_*) = *π _ij_*, the above formula can be written as follows:

$$logit(\pi_{ij})=\beta_{0}+\beta_{1}x_{ij}+\xi_{ij}.$$

The GLMM that furthermore accounts for the patient pairing incorporates additionally a random intercept for each patient *i*:

$$E(Y_{ij}|\beta_{0},\beta_{1},\gamma_{i},\xi_{ij})=logit^{-1}(\beta_{0}+\beta_{1}x_{ij}+\gamma_{i}+\xi_{ij}),$$

where $\gamma_{i}∼N(0,\sigma_{\gamma }^{2}).$

We set up the model formulas and contrast matrix using the createFormula() and createContrast() functions from the *diffcyt* package. These functions create the model formulas and contrast matrix in the required format for the *diffcyt* testing functions. For more details, see ?createFormula and ?createContrast, or refer to the extended documentation in the *diffcyt* vignette ( diffcyt workflow).

ei <- metadata(daf)$experiment_info 
(da_formula1 <- createFormula(ei,                     
    cols_fixed = "condition",      
    cols_random = "sample_id"))    

## $formula
## y ~ condition + (1 | sample_id)
## <environment: 0x7fab710b3b90>
##
## $data
##    condition sample_id
## 1      BCRXL    BCRXL1
## 2        Ref      Ref1
## 3      BCRXL    BCRXL2
## 4        Ref      Ref2
## 5      BCRXL    BCRXL3
## 6        Ref      Ref3
## 7      BCRXL    BCRXL4
## 8        Ref      Ref4
## 9      BCRXL    BCRXL5
## 10       Ref      Ref5
## 11     BCRXL    BCRXL6
## 12       Ref      Ref6
## 13     BCRXL    BCRXL7
## 14       Ref      Ref7
## 15     BCRXL    BCRXL8
## 16       Ref      Ref8
##
## $random_terms
## [1] TRUE

(da_formula2 <- createFormula(ei,               
    cols_fixed = "condition",                   
    cols_random = c("sample_id", "patient_id")))

## $formula
## y ~ condition + (1 | sample_id) + (1 | patient_id)
## <environment: 0x7fab7964e468>
##
## $data
##    condition sample_id patient_id
## 1      BCRXL    BCRXL1   Patient1
## 2        Ref      Ref1   Patient1
## 3      BCRXL    BCRXL2   Patient2
## 4        Ref      Ref2   Patient2
## 5      BCRXL    BCRXL3   Patient3
## 6        Ref      Ref3   Patient3
## 7      BCRXL    BCRXL4   Patient4
## 8        Ref      Ref4   Patient4
## 9      BCRXL    BCRXL5   Patient5
## 10       Ref      Ref5   Patient5
## 11     BCRXL    BCRXL6   Patient6
## 12       Ref      Ref6   Patient6
## 13     BCRXL    BCRXL7   Patient7
## 14       Ref      Ref7   Patient7
## 15     BCRXL    BCRXL8   Patient8
## 16       Ref      Ref8   Patient8
##
## $random_terms
## [1] TRUE

contrast <- createContrast(c(0, 1))

The *diffcyt* package provides three methods for differential abundance (DA) analyses, and two methods for differential state (DS) analyses. For an explanation and comparison of the different methods, see 13. Here, we use the DA methodology based on mixed models, which is implemented in the method diffcyt-DA-GLMM. This method can be selected by providing the arguments method = "DA" and method_DA = "diffcyt-DA-GLMM" to the diffcyt() wrapper function (for more details, see ?diffcyt). As our daFrame contains a total of 22 clusterings (1 high-resolution, 19 consensus mergings, and 2 manual mergings), we also specify the clustering of interest via clustering_to_use = "merging1".

da_res1 <- diffcyt(daf,                                            
    formula = da_formula1, contrast = contrast,                    
    analysis_type = "DA", method_DA = "diffcyt-DA-GLMM",           
    clustering_to_use = "merging1", verbose = FALSE)               
da_res2 <- diffcyt(daf,                                            
    formula = da_formula2, contrast = contrast,
    analysis_type = "DA", method_DA = "diffcyt-DA-GLMM",           
    clustering_to_use = "merging1", verbose = FALSE)               

The *diffcyt* output consists of a list containing several *SummarizedExperiment* objects. The differential test results are stored in the rowData slot of the results object res, and can be accessed using the rowData() accessor function from the *SummarizedExperimentSummarizedExperiment* package. The results include raw p-values ( p_val) and adjusted p-values ( p_adj) for each cluster (for DA tests) or cluster-marker combination (for DS tests), which can be used to rank the clusters or cluster-marker combinations by their evidence for differential abundance (DA tests) or differential states within cell populations (DS tests).

names(da_res1)

## [1] "res"                           "d_counts"
## [3] "d_medians"                     "d_medians_by_cluster_marker"
## [5] "d_medians_by_sample_marker"

rowData(da_res1$res)

## DataFrame with 8 rows and 3 columns
##                cluster_id                p_val                p_adj
##                  <factor>            <numeric>            <numeric>
## B-cells IgM+ B-cells IgM+   0.0134820784148451   0.0409234214313668
## B-cells IgM- B-cells IgM-  0.00554110806328434   0.0409234214313668
## CD4 T-cells   CD4 T-cells   0.0376717369512436   0.0753434739024872
## CD8 T-cells   CD8 T-cells   0.0153462830367626   0.0409234214313668
## DC                     DC    0.415414330818059    0.476379043464654
## NK cells         NK cells    0.416831663031572    0.476379043464654
## monocytes       monocytes    0.547254574996547    0.547254574996547
## surface-         surface-    0.371991525392299    0.476379043464654

When we count the number of differential findings for both GLMMs specified above, we find that accounting for the patient pairing increases the sensitivity to detect differentially abundant cell populations.

table(rowData(da_res1$res)$p_adj < FDR_cutoff)

##
## FALSE TRUE
##     5    3

table(rowData(da_res2$res)$p_adj < FDR_cutoff)

##
## FALSE TRUE
##     2    6

A summary table displaying the results (raw and adjusted p-values) together with the observed cell population proportions by sample can be generated using *diffcyt* ’s topTable() function. For more details, see ?topTable.

topTable(da_res2, show_props = TRUE, format_vals = TRUE, digits = 2)

## DataFrame with 8 rows and 19 columns
##                cluster_id     p_val     p_adj props_Ref1 props_Ref2
##                  <factor> <numeric> <numeric>  <numeric>  <numeric>
## NK cells         NK cells   4.5e-13   3.6e-12       14.3        9.7
## B-cells IgM- B-cells IgM-   2.2e-11   8.8e-11        1.9        1.3
## B-cells IgM+ B-cells IgM+   3.5e-08   9.2e-08        4.8        2.8
## DC                     DC   7.1e-05   0.00014        0.2        0.9
## CD8 T-cells   CD8 T-cells    0.0012    0.0019       23.7       23.8
## CD4 T-cells   CD4 T-cells    0.0019    0.0025       44.7       49.1
## surface-         surface-      0.22      0.26        2.8        2.5
## monocytes       monocytes      0.26      0.26        7.6        9.9
##              props_Ref3 props_Ref4 props_Ref5 props_Ref6 props_Ref7
##               <numeric>  <numeric>  <numeric>  <numeric>  <numeric>
## NK cells           15.1       14.5       10.2        6.7       22.5
## B-cells IgM-        3.3        1.4        2.5        2.3        2.8
## B-cells IgM+        8.3        4.7        4.4        5.7        4.3
## DC                  1.2        1.2        1.6        0.9        0.9
## CD8 T-cells        15.5       17.6         26       25.3       33.5
## CD4 T-cells        39.7       32.4       38.4       47.3       28.2
## surface-              2        1.4        2.3        3.3        2.1
## monocytes          14.9       26.9       14.5        8.5        5.6
##              props_Ref8 props_BCRXL1 props_BCRXL2 props_BCRXL3
##               <numeric>    <numeric>    <numeric>    <numeric>
## NK cells             11         15.6         11.7         16.6
## B-cells IgM-        2.2          1.1            1            2
## B-cells IgM+        3.8          3.9          1.4          4.1
## DC                  0.9          0.2          0.8          1.5
## CD8 T-cells        34.2         46.1         40.9         26.5
## CD4 T-cells        36.9         26.8         35.8         32.3
## surface-            2.4          1.8            2          2.3
## monocytes           8.5          4.5          6.4         14.7
##              props_BCRXL4 props_BCRXL5 props_BCRXL6 props_BCRXL7
##                 <numeric>    <numeric>    <numeric>    <numeric>
## NK cells             18.3         12.6          6.8         25.5
## B-cells IgM-          1.1          1.5          1.2          2.1
## B-cells IgM+          3.8          3.9            4          2.6
## DC                    1.4          2.1          1.4          1.2
## CD8 T-cells          25.9         28.2         24.6         34.3
## CD4 T-cells          31.8         36.7         44.1         26.8
## surface-              1.9          2.1          2.7          1.8
## monocytes            15.7         12.9         15.2          5.7
##              props_BCRXL8
##                 <numeric>
## NK cells             12.9
## B-cells IgM-          1.7
## B-cells IgM+          2.7
## DC                    1.2
## CD8 T-cells          39.7
## CD4 T-cells          31.5
## surface-              2.4
## monocytes             7.8

**Figure 22.**
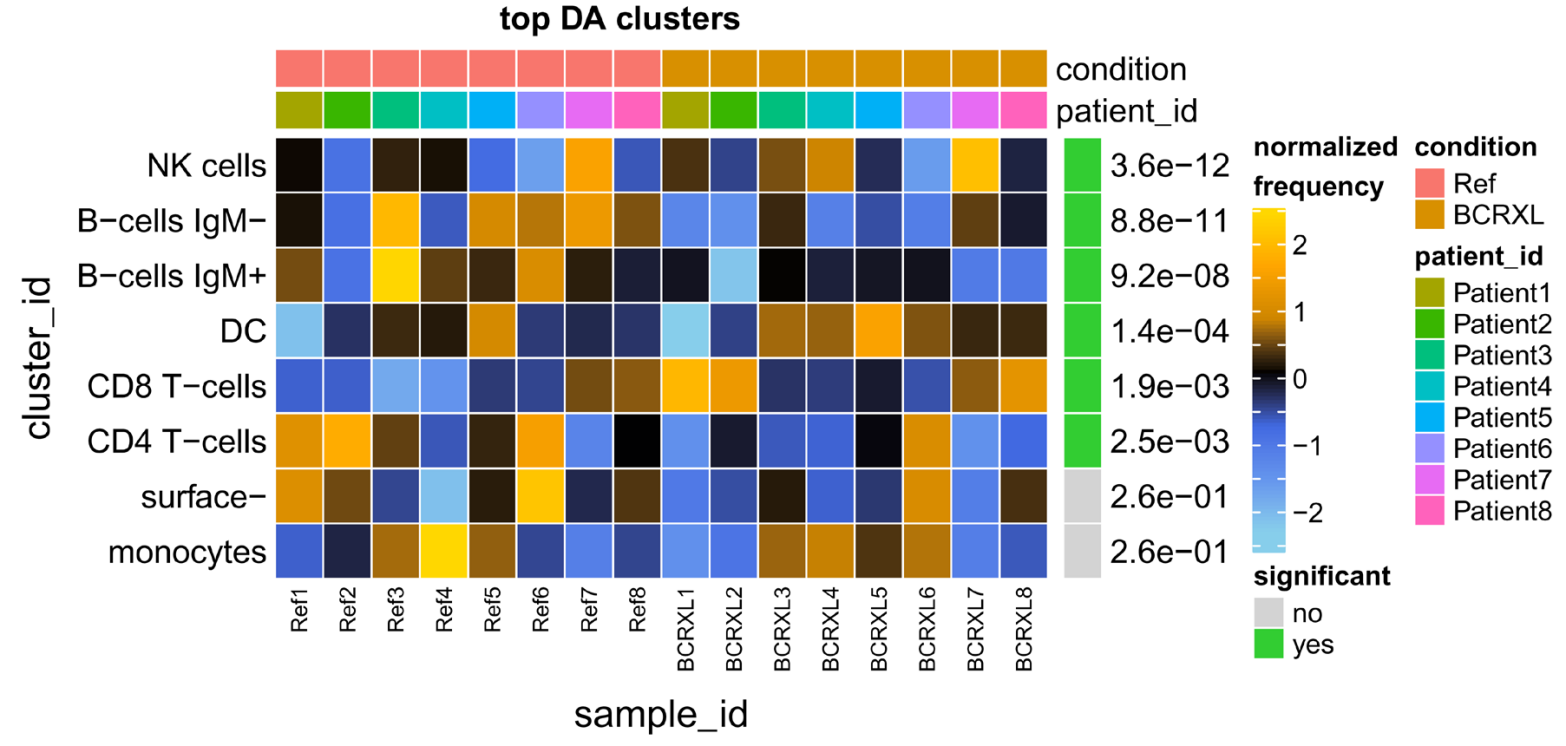
DA test results and normalized proportions for PBMC cell populations in BCR/FcR-XL stimulated and unstimulated conditions. The heat represents arcsine-square-root transformed cell frequencies that were subsequently normalized per cluster (rows) to mean of zero and standard deviation of one. The color of the heat varies from blue indicating relative under-representation to orange indicating relative over-representation. Bars at the top of the heatmap indicate patient IDs and the condition the samples (columns) belong to: red for the unstimulated (Ref) and brown for the stimulated with BCR/FcR-XL (BCRXL) condition. Bar and numbers at the right indicate significant differentially abundant clusters (green) and adjusted p-values. Clusters are sorted according to adjusted p-values, so that the cluster at the top shows the most significant abundance changes between the two conditions.

We use a heatmap to report the differential cell populations ( Figure 22). Proportions are first scaled with the arcsine-square-root transformation (as an alternative to logit that cannot be calculated on ratios of zero or one). Then, z-score normalization is applied to each population to better highlight the relative differences between compared conditions. In addition, the clusters are sorted according to adjusted p-values.

plotDiffHeatmap(daf, da_res2, th = FDR_cutoff, normalize = TRUE, hm1 = FALSE)

### Differential analysis of marker expression stratified by cell population

For this part of the analysis, we calculate the median expression of the 14 signaling markers in each cell population (merged cluster) and sample. These will be used as the response variable *Y _ij_* in the linear model (LM) or linear mixed model (LMM), for which we assume that the median marker expression follows a Gaussian distribution (on the already arcsinh-transformed scale). Analogous to the model above, the linear model is formulated as follows:

$$Y_{ij}=\beta_{0}+\beta_{1}x_{ij}+\epsilon_{ij},$$

where *∈ _ij_ ∼ N*(0, *σ* ^2^), and the mixed model includes a random intercept for each patient:

$$Y_{ij}=\beta_{0}+\beta_{1}x_{ij}+\gamma_{i}+\epsilon_{ij},$$

where $\gamma_{i}∼N(0,\sigma_{\gamma }^{2})$. In the current experiment, we have an intercept (basal level) and a single covariate, *x _ij_*, which is represented as a binary (stimulated/unstimulated) variable. For more complicated designs or batch effects, additional columns of a design matrix can be used.

One drawback of summarizing the protein marker intensity with a median over cells is that all the other characteristics of the distribution, such as bimodality, skewness and variance, are ignored. On the other hand, it results in a simple, easy to interpret approach, which in many cases will be able to detect interesting changes. Another issue that arises from using a summary statistic is the level of uncertainty, which increases as the number of cells used to calculate it decreases. In the statistical modeling, this problem could be partially handled by assigning observation weights (number of cells) to each cluster and sample (parameter weights in the lm and lmer functions used within the *diffcyt* testing functions). However, since each cluster is tested separately, these weights do not account for the differences in size between clusters.

There might be instances of small cell populations for which no cells are observed in some samples or where the number of cells is very low. For clusters absent from a sample (e.g., due to biological variance or insufficient sampling), NAs are introduced because no median expression can be calculated; in the case of few cells, the median may be quite variable. Thus, we apply a filter to remove clusters with very low counts (by default, clusters are kept if they have at least 3 cells in at least half the total number of samples, which is appropriate for a two-group comparison with equal numbers of samples per condition).

**Figure 23.**
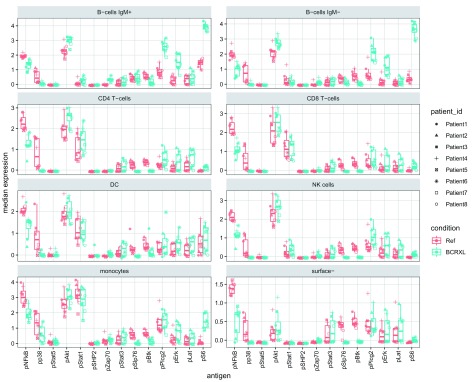
Median (arcsinh-transformed) expression of 14 signaling markers (x-axis) across the 8 identified PBMC cell populations (individual panels). Values for the two conditions are indicated with different colors: red for the unstimulated (Ref) and blue for the stimulated with BCR/FcR-XL (BCRXL) samples. Values for each patient are indicated with different shape. The 8 cell populations are a result of manual merging of the 20 FlowSOM metaclusters.

It is helpful to plot the median expression of all markers in each cluster for each sample colored by condition, to get a rough image of how strong the differences might be ( Figure 23). We do this by combining boxplots and jitter.

p <- plotMedExprs(daf, k = "merging1", facet = "cluster_id",
    shape_by = "patient_id")                                
p$facet$params$ncol <- 2                                    
p                                                           

To present how accounting for the between patient variability with the mixed model increases sensitivity, we also fit a regular linear model. The linear mixed model has a random intercept for each patient.

ds_formula1 <- createFormula(ei, cols_fixed = "condition")
ds_formula2 <- createFormula(ei,                          
    cols_fixed = "condition", cols_random = "patient_id") 

Using *diffcyt*, we calculate differential tests using method diffcyt-DS-LMM, which implements the DS methodology based on mixed models. This method can be selected by providing the arguments method = "DS" and method_DS = "diffcyt-DS-LMM" to the diffcyt() wrapper function. The remaining arguments are the same as for the DA tests.

By accounting for the patient effect, we detect almost twice as many cases of differential signaling compared to the regular linear model.

ds_res1 <- diffcyt(daf,                                            
    formula = ds_formula1, contrast = contrast,                    
    analysis_type = "DS", method_DS = "diffcyt-DS-LMM",            
    clustering_to_use = "merging1", verbose = FALSE)               
table(rowData(ds_res1$res)$p_adj < FDR_cutoff)                     

##
## FALSE TRUE
##    68   44

ds_res2 <- diffcyt(daf,                                            
    formula = ds_formula2, contrast = contrast,                    
    analysis_type = "DS", method_DS = "diffcyt-DS-LMM",            
    clustering_to_use = "merging1", verbose = FALSE)               
table(rowData(ds_res2$res)$p_adj < FDR_cutoff)                     

##
## FALSE TRUE
##    28   84

topTable() is again used to assemble a summary table.

topTable(ds_res2, top_n = 5, order_by = "cluster_id",
    show_meds = TRUE, format_vals = TRUE, digits = 3)

## DataFrame with 5 rows and 20 columns
##                cluster_id marker_id     p_val     p_adj meds_Ref1
##                  <factor>  <factor> <numeric> <numeric> <numeric>
## B-cells IgM+ B-cells IgM+     pNFkB  6.74e-11  3.43e-10     1.964
## B-cells IgM+ B-cells IgM+      pp38    0.0017   0.00355     0.889
## B-cells IgM+ B-cells IgM+    pStat5    0.0348    0.0464    -0.039
## B-cells IgM+ B-cells IgM+      pAkt  6.11e-14  4.56e-13     2.319
## B-cells IgM+ B-cells IgM+    pStat1    0.0714    0.0889    -0.006
##              meds_Ref2 meds_Ref3 meds_Ref4 meds_Ref5 meds_Ref6 meds_Ref7
##              <numeric> <numeric> <numeric> <numeric> <numeric> <numeric>
## B-cells IgM+     1.869     1.773     2.183     1.861     1.953     1.915
## B-cells IgM+     1.113     0.853     0.642     0.126      0.21     0.128
## B-cells IgM+    -0.049    -0.048    -0.024    -0.057    -0.061    -0.054
## B-cells IgM+      2.31     2.269     3.086     1.729     2.024     2.145
## B-cells IgM+     0.064     0.008     0.515    -0.047      0.03    -0.034
##              meds_Ref8 meds_BCRXL1 meds_BCRXL2 meds_BCRXL3 meds_BCRXL4
##              <numeric>   <numeric>   <numeric>   <numeric>   <numeric>
## B-cells IgM+     1.979       1.179        0.88       0.808       1.473
## B-cells IgM+     0.126       0.109      -0.012       0.044       0.245
## B-cells IgM+    -0.053      -0.037      -0.037      -0.029       0.056
## B-cells IgM+     2.603       3.247        2.96       2.951       3.257
## B-cells IgM+     0.191       0.343       0.126       0.242       0.333
##              meds_BCRXL5 meds_BCRXL6 meds_BCRXL7 meds_BCRXL8
##                <numeric>   <numeric>   <numeric>   <numeric>
## B-cells IgM+       1.361       1.725       1.436       1.575
## B-cells IgM+      -0.046       0.083      -0.039      -0.005
## B-cells IgM+      -0.067      -0.015      -0.051      -0.039
## B-cells IgM+       2.382       3.184       2.762       3.144
## B-cells IgM+       -0.01       0.616       -0.05       0.379

**Figure 24.**
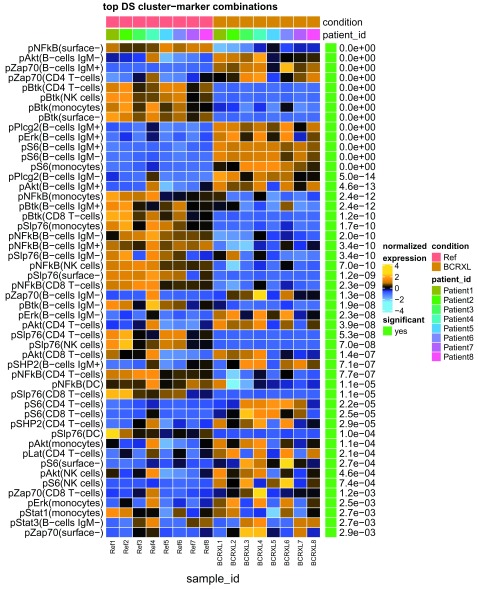
DS test results and normalized expression of signaling markers in PBMC populations in BCR/FcR- XL stimulated and unstimulated conditions. The heat represents median (arcsinh-transformed) marker expression that was subsequently normalized per cluster-marker combination (rows) to mean of zero and standard deviation of one. The color of the heat varies from blue representing relative under-expression to orange representing relative over-expression. Bars at the top of the heatmap indicate patient IDs and the condition the samples (columns) belong to: red for the unstimulated (Ref) and brown for the stimulated with BCR/FcR-XL (BCRXL) condition. Bar and numbers at the right indicate significant differential cluster-marker combinations (green) and adjusted p-values. Cluster-marker combinations are sorted according to adjusted p-value, so that the cluster-marker combinations at the top show the most significant differential signals between the two conditions. Shown are only the top 50 most highly significant cluster-marker combinations.

We use a heatmap to report the differential signals ( Figure 24). Instead of plotting the absolute expression, we display the normalized expression, which better highlights the direction of marker changes. In addition, the cluster-marker combinations are sorted according to adjusted p-value.

plotDiffHeatmap(daf, ds_res2, top_n = 50, order = TRUE,    
    th = FDR_cutoff, normalize = TRUE, hm1 = FALSE)        

### Differential analysis of overall marker expression

The analysis of *overall* expression is analogous to the previous section, except that median marker expression is aggregated from all the cells in a given sample. For this, we generate an artificial clustering with all cells assigned to a single cluster.

daf <- mergeClusters(daf, k = "meta20", id = "merging_all",            
    table = data.frame(old_cluster = seq_len(20), new_cluster = "all"))

**Figure 25.**
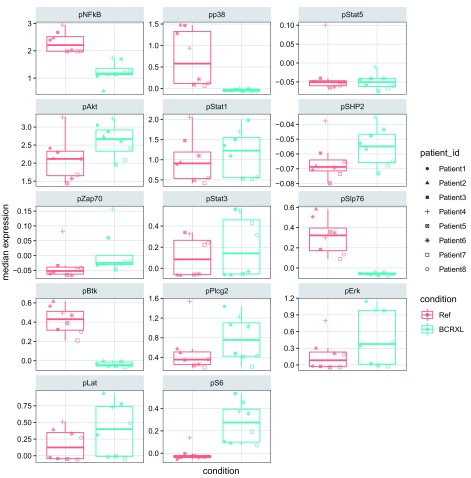
Median (arcsinh-transformed) expression of 14 signaling markers calculated from all the cells in a given sample in the PBMC dataset. Values for the two conditions are indicated with different colors: red for the unstimulated (Ref) and blue for the stimulated with BCR/FcR-XL (BCRXL) samples. Values for each patient are indicated with different shape.

As before, the median expression can be plotted ( Figure 25).

p <- plotMedExprs(daf[, state_markers(daf)], shape_by = "patient_id")
p$facet$params$ncol <- 3                                             
p                                                                    

Similar to the analysis above, we identify more markers being differentially expressed with the LMM, which accounts for the between patient variability.

# fit linear model                                                 
ds_res3 <- diffcyt(daf,                                            
    formula = ds_formula1, contrast = contrast,                    
    analysis_type = "DS", method_DS = "diffcyt-DS-LMM",            
    clustering_to_use = "merging_all", verbose = FALSE)            
                                                                   
# fit linear mixed model with patient ID as random effect          
ds_res4 <- diffcyt(daf,                                            
    formula = ds_formula2, contrast = contrast,                    
    analysis_type = "DS", method_DS = "diffcyt-DS-LMM",            
    clustering_to_use = "merging_all", verbose = FALSE)            
                                                                   
table(rowData(ds_res3$res)$p_adj < FDR_cutoff)                     

##
## FALSE TRUE
##     9    5

table(rowData(ds_res4$res)$p_adj < FDR_cutoff)

##
## FALSE TRUE
##     2   12

**Figure 26.**
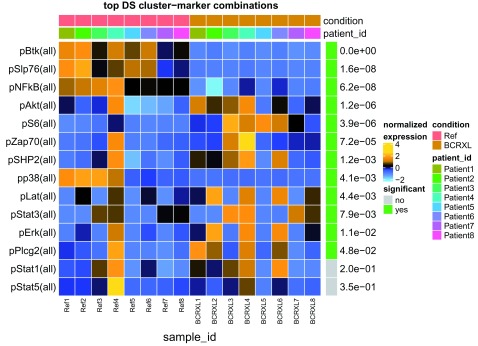
DS test results and normalized expression of signaling markers calculated over all cells in PBMC populations in BCR/FcR-XL stimulated and unstimulated conditions. The heat represents median (arcsinh-transformed) marker expression that was subsequently normalized per marker (rows) to mean of zero and standard deviation of one. The color of the heat varies from blue representing relative under-expression to orange representing relative over-expression. Bars at the top of the heatmap indicate patient IDs and the condition the samples (columns) belong to: red for the unstimulated (Ref) and brown for the stimulated with BCR/FcR-XL (BCRXL) condition. Bar and numbers at the right indicate significant differential markers (green) and adjusted p-values. Markers are sorted according to adjusted p-value, so that the marker at the top shows the most significant change between the two conditions.

As before, we create a summary table and heatmap displaying the results ( Figure 26).

topTable(ds_res4, top_n = 5, order_by = "p_adj",     
    show_meds = TRUE, format_vals = TRUE, digits = 3)

## DataFrame with 5 rows and 20 columns
##     cluster_id marker_id     p_val     p_adj meds_Ref1 meds_Ref2 meds_Ref3
##       <factor>  <factor> <numeric> <numeric> <numeric> <numeric> <numeric>
## all        all      pBtk         0         0     0.566     0.615     0.323
## all        all    pSlp76  2.22e-09  1.56e-08     0.506     0.582     0.184
## all        all     pNFkB  1.33e-08  6.21e-08     2.392     2.469      2.67
## all        all      pAkt  3.55e-07  1.24e-06     2.416     2.122       2.3
## all        all       pS6   1.4e-06  3.92e-06    -0.055    -0.039    -0.002
##     meds_Ref4 meds_Ref5 meds_Ref6 meds_Ref7 meds_Ref8 meds_BCRXL1
##     <numeric> <numeric> <numeric> <numeric> <numeric>   <numeric>
## all     0.494      0.39     0.471     0.209     0.297      -0.041
## all     0.297     0.358     0.347      0.09     0.133      -0.051
## all      2.94     1.979     2.025      1.98     1.985        1.07
## all     3.273     1.443     1.573      1.68     2.114       3.053
## all     0.138    -0.025    -0.038    -0.032    -0.029       0.102
##     meds_BCRXL2 meds_BCRXL3 meds_BCRXL4 meds_BCRXL5 meds_BCRXL6
##       <numeric>   <numeric>   <numeric>   <numeric>   <numeric>
## all      -0.007      -0.055      -0.016      -0.054      -0.009
## all      -0.058      -0.062      -0.038      -0.071      -0.045
## all        0.52       1.144        1.74       1.143       1.695
## all       2.727       2.876       3.242       1.958       2.607
## all        0.09       0.534       0.373       0.454       0.356
##     meds_BCRXL7 meds_BCRXL8
##       <numeric>   <numeric>
## all       -0.07      -0.051
## all      -0.074      -0.063
## all       1.195       1.245
## all       2.075       2.416
## all       0.193        0.07

plotDiffHeatmap(daf, ds_res4, all = TRUE, hm1 = FALSE)

## Obtaining higher resolution

In the proposed workflow, we concentrated on identification of the main cell types in PBMCs. Our goal was to identify around 6 main cell types. Following the over-clustering strategy, we have chosen to perform the SOM clustering into 100 (by default) groups followed by consensus clustering into 20 groups, from which we could annotate 8 cell types. These 8 cell types were then used in the differential analysis.

If the number of expected cell types is higher, the user can increase the size of the SOM grid in *CATALYST*’s cluster() function using the xdim and ydim arguments and increase the maximum number of consensus clusters via the maxK argument.

One could also use a strategy based on subsequent clustering of identified clusters, which we refer to as *reclustering*. In the starting step, one uses the presented workflow to identify the main cell types. In the following steps, the same clustering workflow is applied individually to the cell populations for which more resolution is desired. Restricting to one subpopulation at a time results in easier cluster annotation. The differential analysis can be applied to the final clusters in the same way as described in the workflow assuming tables with cell counts and median marker expression are available.

The differential analysis could also be conducted on the unmerged (20) consensus clusters and the manual annotation could be done at the end.

## Discussion

In this workflow, we have presented a pipeline for diverse differential analyses of HDCyto datasets. First, we highlight quality control steps, where aggregate characteristics of the samples are visualized (e.g., an MDS plot), allowing for verification of the experimental design, detection of batch effects and outlying samples. Next, cell population identification was carried out via clustering, which forms the basis for subsequent differential analyses of cell population abundance, differential marker expression within a population or overall marker expression differences. The approaches to differential analyses proposed here are very general and thus able to model complex experimental designs via design matrices, such as factorial experiments, paired experiments or adjustment for batch effects. We have presented a range of visualizations that help in understanding the data and reporting the results of clustering and differential analyses.

Clustering is one of the most challenging steps in the workflow, and its accuracy is critical to the downstream differential analyses. Getting the right resolution of clusters is crucial, since there can be situations where a biologically meaningful cell population may be differentially enriched between conditions, but in an automatic clustering, was combined with another cell population that behaves differently. We have shown that some level of over-clustering is convenient for detecting meaningful cell populations, since automatic detection of the number of natural clusters is difficult ^15^. However, there are tradeoffs between the resolution of clustering and the labor involved in aggregating them to biologically meaningful clusters. Overall, we take an interactive but flexible algorithm-guided approach together with subject-area experts to arrive at sensible cell populations. In particular, we rely on various visualizations, such as dendrograms, heatmaps, UMAP embeddings or other dimension reduction techniques to guide us in the process. Alternative strategies could be combined with the statistical inference we present, such as over-clustering combined with data-driven aggregation to the optimal resolution.

While we have a good understanding of how computational algorithms recapitulate manual gating in high dimensions ^15^, one of the open areas of research remains how to best cluster *across* samples. The data analyzed here ^8, 21^ was generated using sample barcoding; this strategy reduces inter-sample variability, since all samples are exposed to the same antibody cocktail and measured in a single acquisition ^26^. Thus, the range of marker expression for each channel should, in principle, be within a similar range across samples.

In our approach, we aggregated all cells together before clustering. Because of this aggregation, the clustering is blind to the sample labels, and thus in principle, does not bias the downstream statistical inferences. Moreover, we directly obtain consistent clustering between samples. However, some challenges may arise when there are substantial differences in numbers of cells in samples. There is a risk that larger samples may drive the final clustering results. A simple solution to this problem could be ensuring that each sample contributes an equal amount of cells into the clustering analysis. This could be done by sampling an equal number of cells from each sample. However, there are two main drawbacks of this strategy. First, a substantial amount of data (cells) may be removed from the analysis if there are samples with few cells, thus resulting in information loss. Second, during downsampling, some of the smaller populations may become under-represented or even skipped. An alternative would be to cluster within each sample and then aggregate a collection of metaclusters across samples ^45^. A recent approach, called PAC-MAN ^46^, uses a combination of high-dimensional density estimation, hierarchical clustering and network inference and comparison to extract clusters across samples, with a possibility to handle batch effects.

Additional challenges may arise when combining data from different instrument acquisitions and additional preprocessing treatments may need to be applied. Despite adjustments through bead-based normalization ^25^, the observed marker expression may be affected by the varying efficiency of antibody binding in each batch and by the ion detection sensitivity after machine calibration. Beyond normalization, other strategies have been proposed, such as equalizing the dynamic range between batches for each marker (e.g., normalization to the 0–1 range, z-scores, quantile normalization), the use of warping functions to eliminate non-linear distortions (see the *cydar* vignette), landmark-based normalization ^47^, or learning marker distribution shifts between the batches based on a manually gated reference cell type and using it to correct marker expression for the whole dataset ^9^.

Alternatively, one could consider batch-wise clustering of samples. On the other hand, to be able to use those results, one still needs to match cell populations across batches. The matching could be done manually, or with automated approaches developed for flow cytometry ^45^. However, a comprehensive evaluation of these approaches and their effect on downstream analyses is still missing. Overall, we expect that as a general rule, including batch parameters (or other covariates) in the linear modeling helps to mitigate the problem. In single cell RNA sequencing data, several methods to “align” samples have been proposed ^48– 51^ and these strategies may have applications to cytometry data.

We presented a classical statistical approach where preprocessing of the HDCyto data leads to tables of summaries (e.g., cell counts) or aggregated measurements (e.g., cluster-specific signals) for each sample, which become the input to a statistical model. Of course, there are a variety of alternative computational approaches available to the user. We have mentioned *Citrus* and *CellCnn*, which are both machine-learning approaches that fit a reverse model to ours (i.e., phenotype of interest as the response variable).

Another set of methods ( *MIMOSA* and *COMPASS*), based on a Bayesian hierarchical framework, was proposed in the vaccine development field, where the antigen-specific T-cell response to stimulation for each subject is modeled using mixtures of beta-binomial or Dirichlet-multinomial distributions ^52, 53^. These strategies bear similarity to the mixed models applied for differential abundance in this workflow while handling over-dispersion due to subject-to-subject variability.

Neither of these approaches are directly able to account for batch effects or complicated designs. However, they may have advantages in the search for rare distinguishing populations, which could be used together with our framework for formal statistical testing.

One of the main goals of this workflow was to highlight how a model-based approach is able to handle complex experimental designs. This becomes important in many experimental situations where covariates (e.g., age, gender, batch) may affect the observed HDCyto data. Thus, the classical regression framework allows also to flexibly test situations well beyond two-group differences. Of course, alternatives exist for two group comparisons, such as the nonparametric Mann-Whitney-Wilcoxon test ^6^, which makes no assumptions about normality of the data, or the Student’s t-test ^7^ and its variations, such as the paired t-test.

We note that the LM, LMM and GLMM may perform poorly for extremely small samples. Here, solutions similar to those widely accepted in transcriptomics that share information over variance parameters ^54– 56^ can instead be leveraged. We have recently implemented methods based on these ideas in the *diffcyt* package ^13^. Similarly, the *cydar* ^10^ package performs differential abundance analysis (on “hypersphere” counts) using the generalized linear modeling capabilities of *edgeR* ^11^. For a detailed explanation and comparison of these alternative approaches, see 13.

In the differential marker expression analysis, we compare the median marker expression between samples. While in many cases this approach is sufficient to detect interesting changes, by summarizing marker expression over cells to a single value we ignore all the other characteristics of the expression distribution, such as bimodality, skewness and variance, which may be relevant in some studies. Thus, it may be interesting to extend our comparisons to the whole marker distributions, instead of just changes in the medians.

The approach presented in this workflow is not fully automated due to the cluster merging, annotating, and extensive exploratory data analysis steps. In general, our philosophy is that fully automated analyses are to be avoided, but rather a battery of diagnostic checks can be designed, as we have promoted here. Cluster annotation remains a manual step in many other approaches as well. Recently, a tool was proposed for consistent characterization of cell subsets using marker enrichment modeling (MEM) ^57^.

To keep the analysis of this workflow reproducible, one needs to define a random seed when running *FlowSOM*, t-SNE and UMAP. This is especially important in the clustering step, where the order of clusters may change with different seeds, and the cluster merging needs to be matched to the seed used. (Note that as mentioned at the start of the workflow, we also use the function RNGversion() to ensure backward compatibility with earlier versions of the workflow, due to changes to the default random number generation methods in R.)

## Software availability

All software packages used in this workflow are publicly available from the Comprehensive R Archive Network ( https://cran.r-project.org) or the Bioconductor project ( http://bioconductor.org). The specific version numbers of the packages used are shown below, along with the version of the R installation.

Version numbers of all the Bioconductor packages correspond to the release version 3.9 of the Bioconductor project. Note that Bioconductor releases new versions of packages every 6 months and it is generally good practice to use the latest versions.

Users can install all required packages and execute the workflow by following the instructions at https://www.bioconductor.org/help/workflows/cytofWorkflow.

sessionInfo()

## R version 3.6.0 (2019-04-26)
## Platform: x86_64-apple-darwin15.6.0 (64-bit)
## Running under: macOS High Sierra 10.13.6
##
## Matrix products: default
## BLAS:   /System/Library/Frameworks/Accelerate.framework/Versions/A/Frameworks/vecLib.framework/Versions/
## LAPACK: /Library/Frameworks/R.framework/Versions/3.6/Resources/lib/libRlapack.dylib
##
## Random number generation:
##  RNG:     Mersenne-Twister
##  Normal:  Inversion
##  Sample:  Rounding
##
## locale:
## [1] en_US.UTF-8/en_US.UTF-8/en_US.UTF-8/C/en_US.UTF-8/en_US.UTF-8
##
## attached base packages:
## [1] stats4    parallel stats      graphics grDevices utils      datasets
## [8] methods   base
##
## other attached packages:
##  [1] HDCytoData_1.4.0              SummarizedExperiment_1.14.0
##  [3] DelayedArray_0.10.0           BiocParallel_1.18.0
##  [5] matrixStats_0.54.0            Biobase_2.44.0
##  [7] GenomicRanges_1.36.0          GenomeInfoDb_1.20.0
##  [9] IRanges_2.18.0                S4Vectors_0.22.0
## [11] ExperimentHub_1.10.0          AnnotationHub_2.16.0
## [13] BiocFileCache_1.8.0           dbplyr_1.4.0
## [15] BiocGenerics_0.30.0           readxl_1.3.1
## [17] flowCore_1.50.0               diffcyt_1.4.3
## [19] cowplot_0.9.4                 ggplot2_3.1.1
## [21] CATALYST_1.8.3                knitr_1.22
## [23] BiocStyle_2.12.0
##
## loaded via a namespace (and not attached):
##   [1] circlize_0.4.6                drc_3.0-1
##   [3] plyr_1.8.4                    igraph_1.2.4.1
##   [5] ConsensusClusterPlus_1.48.0   lazyeval_0.2.2
##   [7] shinydashboard_0.7.1          splines_3.6.0
##   [9] scater_1.12.0                 TH.data_1.0-10
##  [11] digest_0.6.18                 htmltools_0.3.6
##  [13] viridis_0.5.1                 memoise_1.1.0
##  [15] magrittr_1.5                  cluster_2.0.8
##  [17] openxlsx_4.1.0                limma_3.40.0
##  [19] ComplexHeatmap_2.0.0          RcppParallel_4.4.2
##  [21] sandwich_2.5-1                colorspace_1.4-1
##  [23] rappdirs_0.3.1                blob_1.1.1
##  [25] rrcov_1.4-7                   ggrepel_0.8.1
##  [27] haven_2.1.0                   xfun_0.7
##  [29] dplyr_0.8.0.1                 crayon_1.3.4
##  [31] RCurl_1.95-4.12               jsonlite_1.6
##  [33] graph_1.62.0                  lme4_1.1-21
##  [35] survival_2.44-1.1             zoo_1.8-5
##  [37] glue_1.3.1                    gtable_0.3.0
##  [39] nnls_1.4                      zlibbioc_1.30.0
##  [41] XVector_0.24.0                GetoptLong_0.1.7
##  [43] car_3.0-2                     BiocSingular_1.0.0
##  [45] shape_1.4.4                   SingleCellExperiment_1.6.0
##  [47] DEoptimR_1.0-8                abind_1.4-5
##  [49] scales_1.0.0                  mvtnorm_1.0-10
##  [51] DBI_1.0.0                     edgeR_3.26.1
##  [53] Rcpp_1.0.1                    plotrix_3.7-5
##  [55] viridisLite_0.3.0             xtable_1.8-4
##  [57] clue_0.3-57                   bit_1.1-14
##  [59] foreign_0.8-71                rsvd_1.0.0
##  [61] FlowSOM_1.16.0                tsne_0.1-3
##  [63] DT_0.6                        htmlwidgets_1.3
##  [65] httr_1.4.0                    RColorBrewer_1.1-2
##  [67] pkgconfig_2.0.2               XML_3.98-1.19
##  [69] uwot_0.1.3                    locfit_1.5-9.1
##  [71] labeling_0.3                  AnnotationDbi_1.46.0
##  [73] tidyselect_0.2.5              rlang_0.3.4
##  [75] reshape2_1.4.3                later_0.8.0
##  [77] munsell_0.5.0                 cellranger_1.1.0
##  [79] tools_3.6.0                   RSQLite_2.1.1
##  [81] ggridges_0.5.1                evaluate_0.13
##  [83] shinyBS_0.61                  stringr_1.4.0
##  [85] yaml_2.2.0                    bit64_0.9-7
##  [87] zip_2.0.1                     robustbase_0.93-5
##  [89] purrr_0.3.2                   nlme_3.1-139
##  [91] mime_0.6                      compiler_3.6.0
##  [93] interactiveDisplayBase_1.22.0 beeswarm_0.2.3
##  [95] plotly_4.9.0                  curl_3.3
##  [97] png_0.1-7                     tibble_2.1.1
##  [99] pcaPP_1.9-73                  stringi_1.4.3
## [101] highr_0.8                     RSpectra_0.14-0
## [103] forcats_0.4.0                 lattice_0.20-38
## [105] Matrix_1.2-17                 nloptr_1.2.1
## [107] shinyjs_1.0                   pillar_1.4.0
## [109] BiocManager_1.30.4            GlobalOptions_0.1.0
## [111] RcppAnnoy_0.0.12              BiocNeighbors_1.2.0
## [113] data.table_1.12.2             bitops_1.0-6
## [115] irlba_2.3.3                   corpcor_1.6.9
## [117] httpuv_1.5.1                  R6_2.4.0
## [119] bookdown_0.10                 promises_1.0.1
## [121] gridExtra_2.3                 rio_0.5.16
## [123] vipor_0.4.5                   codetools_0.2-16
## [125] boot_1.3-22                   MASS_7.3-51.4
## [127] gtools_3.8.1                  assertthat_0.2.1
## [129] rjson_0.2.20                  withr_2.1.2
## [131] multcomp_1.4-10               GenomeInfoDbData_1.2.1
## [133] hms_0.4.2                     grid_3.6.0
## [135] minqa_1.2.4                   tidyr_0.8.3
## [137] rmarkdown_1.12                DelayedMatrixStats_1.6.0
## [139] carData_3.0-2                 Rtsne_0.15
## [141] shiny_1.3.2                   tinytex_0.13
## [143] ggbeeswarm_0.6.0
