# Supplementary material for: CyTOF workflow: differential discovery in high-throughput high-dimensional cytometry datasets
Source: F1000Res. 2019 May 24;6:748. Originally published 2017 May 26. [Version 3] doi: 10.12688/f1000research.11622.3 (PMC5473464; doi:10.12688/f1000research.11622.3)
Supplement: Peer review history

# Peer review: Reviewer response for version 1

**DOI:** 10.5256/f1000research.12553.r23051

**Published:** 2017-06-08

**Authors:** Greg Finak, Raphael Gottardo

**Affiliations:** 1 Vaccine and Infectious Disease Division (VIDD), Fred Hutchinson Cancer Research Center, Seattle, WA, USA

Nowicka and colleagues present a detailed workflow for analyzing high dimensional cytometry data using open source tools within the Bioconductor framework. 

 The paper provides a clear path for analyzing high dimensional cytometry data, with biomarker discovery in mind, starting from raw data, through preprocessing, population discovery, annotation, and differential abundance analysis. 

 Two particular strengths of the proposed approach are i) the decision to use expert-guided merging of cell populations, and ii) the model-based differential abundance analysis of cell populations. 

 The proposed visualization and summaries of the data make i) straightforward to follow and justify, and adequate alternatives are provided and shown to perform equally well in instances where manual merging of many clusters would be cumbersome. 

 The modeling of cell population counts, rather than proportions, is an approach that we strongly support, and the use of logistic regression with mixed effects is a natural approach that is probably insufficiently appreciated by the community at large. That said, some of the methods proposed in the workflow have been in use in the vaccine development field for some time and should be appropriately cited.

 Specifically, in the section "Visual representation with tSNE", the authors promote coloring individual cells on a tSNE map by expression level, and later still, stratifying by condition (Fig. 11). We point the authors the article by Lin et al. ^1^,  where a very similar approach, using bioconductor tools, is undertaken to identify and visualize polyfunctional Ag-specific T-cells.

 The discussion of existing methodological approaches to identify cytometry biomarkers associated with outcome and the discussion of modeling cell counts in favor of modeling of proportions is important, but should also reference existing work in the vaccine development field. Our group has done substantial work in this area, developing count-based models for antigen-specific T-cell response to stimulation[ref-2 ^,^^3^, the latter of which identified a novel biomarker of infection risk in an HIV clinical trial.

 While these methods do not account for covariates, they are relevant to the discussion since they utilize the Beta-binomial and Dirichlet-Multinomial distributions in a Bayesian formulation to handle over-dispersion due to subject-to-subject variability (an alternative to mixed effects modeling), and warrant mention here.

 Some additional minor points: the citation of flowCore (p5) should reference the journal publication describing the software ^4^, since it is available, rather than the software vignette.

 Finally, note that flowCore is not used for analysis (p4),  which is this context we take to mean clustering or gating, but rather is an infrastructure package that will read, write and transform cytometry data, as well as defining gate objects.

 The core infrastructure for actually performing data-driven gating in Bioconductor is implemented in packages like flowWorkspace (Finak G, Jiang M, Gottardo R. flowWorkspace: Infrastructure for representing and interacting with the gated cytometry. 2011.) and  openCyto ^5^.

 The citations above should be added and updated for completeness and clarity.

 Other than the above, the article is scientifically sound and the conclusions are justified by the data.

We have read this submission. We believe that we have an appropriate level of expertise to confirm that it is of an acceptable scientific standard.

## References

[1] LinLFrelingerJJiangWFinakGSeshadriCBartPAPantaleoGMcElrathJDeRosaSGottardoR: Identification and visualization of multidimensional antigen-specific T-cell populations in polychromatic cytometry data. *Cytometry A*.2015;87(7) : 10.1002/cyto.a.22623 675-82 10.1002/cyto.a.22623 25908275PMC4482785

[2] FinakGMcDavidAChattopadhyayPDominguezMDe RosaSRoedererMGottardoR: Mixture models for single-cell assays with applications to vaccine studies. *Biostatistics*.2014;15(1) : 10.1093/biostatistics/kxt024 87-101 10.1093/biostatistics/kxt024 23887981PMC3862207

[3] LinLFinakGUsheyKSeshadriCHawnTRFrahmNScribaTJMahomedHHanekomWBartPAPantaleoGTomarasGDRerks-NgarmSKaewkungwalJNitayaphanSPitisuttithumPMichaelNLKimJHRobbMLO'ConnellRJKarasavvasNGilbertPC De RosaSMcElrathMJGottardoR: COMPASS identifies T-cell subsets correlated with clinical outcomes. *Nat Biotechnol*.2015;33(6) : 10.1038/nbt.3187 610-6 10.1038/nbt.3187 26006008PMC4569006

[4] HahneFLeMeurNBrinkmanRREllisBHaalandPSarkarDSpidlenJStrainEGentlemanR: flowCore: a Bioconductor package for high throughput flow cytometry. *BMC Bioinformatics*.2009;10: 10.1186/1471-2105-10-106 106 10.1186/1471-2105-10-106 19358741PMC2684747

[5] FinakGFrelingerJJiangWNewellEWRameyJDavisMMKalamsSADe RosaSCGottardoR: OpenCyto: an open source infrastructure for scalable, robust, reproducible, and automated, end-to-end flow cytometry data analysis. *PLoS Comput Biol*.2014;10(8) : 10.1371/journal.pcbi.1003806 e1003806 10.1371/journal.pcbi.1003806 25167361PMC4148203

# Response

**Published:** 2017-10-31

**Authors:** Mark Robinson

**Affiliations:** University of Zurich, Switzerland

Thank you for taking the time to read and review our paper. Following the suggestion of the reviewer, we have incorporated the missing references, including the reference to Lin et al. [1] in the "Visual representation with tSNE" section, and references to MIMOSA [2] and COMPASS [3] methods in the "Discussion" section. We have also fixed references in the "Data preprocessing" section, including the reference to the flowCore package [4]. We have clarified that the packages that can be used for cell gating are flowWorkspace [5] and openCyto [6].

 References

 [1] Lin Lin, Jacob Frelinger, Wenxin Jiang, Greg Finak, Chetan Seshadri, Pierre-Alexandre Bart, Giuseppe Pantaleo, Julie McElrath, Steve DeRosa, and Raphael Gottardo. *Identification and visualization of multidimensional antigen-specific T-cell populations in polychromatic cytometry data*. **Cytometry Part A**, 87(7):675–682, 2015.

 [2] Greg Finak, Andrew McDavid, Pratip Chattopadhyay, Maria Dominguez, Steve De Rosa, Mario Roederer, and Raphael Gottardo. *Mixture models for single-cell assays with applications to vaccine studies*. **Biostatistics**, 15(1):87–101, 2014.

 [3] Lin Lin, Greg Finak, Kevin Ushey, Chetan Seshadri, Thomas R Hawn, Nicole Frahm, Thomas J Scriba, Hassan Mahomed, Willem Hanekom, Pierre-Alexandre Bart, Giuseppe Pantaleo, Georgia D Tomaras, Supachai Rerks-Ngarm, Jaranit Kaewkungwal, Sorachai Nitayaphan, Punnee Pitisuttithum, Nelson L Michael, Jerome H Kim, Merlin L Robb, Robert J O’Connell, Nicos Karasavvas, Peter Gilbert, Stephen C De Rosa, M Juliana McElrath, and Raphael Gottardo. *COMPASS identifies T-cell subsets correlated with clinical outcomes*. **Nat Biotech**, 33(6):610–616, jun 2015.

 [4] Florian Hahne, Nolwenn LeMeur, Ryan R Brinkman, Byron Ellis, Perry Haaland, Deepayan Sarkar, Josef Spidlen, Errol Strain, and Robert Gentleman. *flowCore: a Bioconductor package for high throughput flow cytometry*. **BMC Bioinformatics**, 10(1):106, apr 2009.

 [5] Greg Finak and Mike Jiang. *flowWorkspace: Infrastructure for representing and interacting with the gated cytometry*, 2011. R package version 3.24.4.

 [6] Greg Finak, Jacob Frelinger, Wenxin Jiang, Evan W EW Newell, John Ramey, Mark MM Davis, SA Spyros a Kalams, SC Stephen C De Rosa, and Raphael Gottardo. OpenCyto: An *Open Source Infrastructure for Scalable, Robust, Reproducible, and Automated, End-to-End Flow Cytometry Data Analysis*. **PLoS Computational Biology**, 10(8):e1003806, 2014.

# Peer review: Reviewer response for version 1

**DOI:** 10.5256/f1000research.12553.r23055

**Published:** 2017-06-06

**Authors:** Aaron T. L. Lun, John C. Marioni

**Affiliations:** 1 CRUK (Cancer Research UK) Cambridge Institute, University of Cambridge, Cambridge, UK; 2 EMBL-European Bioinformatics Institute (EMBL-EBI), Wellcome Genome Campus, Cambridge, UK; 3 Wellcome Trust Sanger Institute, Wellcome Genome Campus, Cambridge, UK

Nowicka et al. describe a comprehensive workflow for a multi-sample analysis of a mass cytometry data set. They provide methods and guidelines for data processing and quality control, clustering and interpretation/visualization of the clusters. They also describe the application of statistical methods for differential analyses within clusters. The article is clear and well-written, with some opportunities for improvement that we have listed below. Overall, this will be useful resource for people looking to use R/Bioconductor for cytometry data analysis.

**MAJOR COMMENTS:**

1. The principle of pooling samples prior to clustering is important to ensure that the clustering is blind to the sample labels, and thus does not bias the downstream statistical inferences. However, in data sets where the number of cells is highly variable across samples, larger samples may drive the final clustering result. The authors may consider downweighting cells from larger samples during the clustering procedure, to ensure that each sample contributes equally to the outcome.
2. In what scenarios is the NRS useful? In a mass cytometry experiment, the panel is explicitly designed to interrogate markers of interest, so outside of quality control it makes little sense to discard them in the analysis. Indeed, low variance contributions in PCA does not mean that the marker is not relevant, e.g., if it marks a small population.
3. If the expected number of cell types is not known in advance, how many metaclusters should be chosen? In very heterogeneous populations, it is easy to imagine that there may well be more than 20 distinct cell subpopulations.
4. In the discussion, the authors state that "Overall, we expect that as a general rule, including batch parameters (or other covariates) in the linear modeling largely mitigates the problem." This is true to some extent, but will not protect the clustering from batch effects. If the batch effect shifts the intensity distribution between batches, it is possible that a subpopulation in samples of one batch is clustered with the wrong subpopulation in samples of another batch. The counts or median intensities of the cluster are inherently compromised and cannot be fixed by blocking on the batch effect in the model. In other words; when testing for changes in abundance in mass cytometry, the cells are analogous to individual reads in a transcriptomics experiment, while the vector of intensities is analogous to the genic region in which reads are counted. If the batch effect is affecting the intensities, it is analogous to changes in the definition of the genes between batches.
5. The authors mention using observation weights to describe the uncertainty of the median intensities when testing for differential expression of markers. We note that we have also used this approach in cydar, and it seems to work well. For differential expression of markers within each cluster, the differences in size between clusters are largely irrelevant - all else being equal, if clusters are small, the medians should be more variable, and this should be considered by the inference machinery when computing p-values.

**MINOR COMMENTS:**

- A mention should be made of the fact that fsApply combines the intensity matrices from all data sets; this was not obvious from the code.
- There are many ways to do hypothesis testing in GLMMs, with options ranging from Wald Z-tests, LRTs and parametric bootstrapping/MCMC. Some words on what glht actually does would be useful.
- Some of the figure captions could be explained in more detail. For example, the numbers and colouring of the entries of the heatmap in Figure 4 are presumably the median marker intensities, but this should be explicitly stated.
- Some minor typographical errors: " (Angerer et al., 2016))", "use the flowCore [package]."
- The PBMC data set is described as "12 different stimulation conditions", but presumably only one was actually used (BCR/FcR-XL). This could be clarified.

We have read this submission. We believe that we have an appropriate level of expertise to confirm that it is of an acceptable scientific standard.

# Response

**Published:** 2017-10-31

**Authors:** Mark Robinson

**Affiliations:** University of Zurich, Switzerland

Thank you for taking the time to read and review our paper.

 MAJOR POINTS:

1. As far as we understand, downweighting of cells (e.g., from larger samples) is not currently possible with FlowSOM. We think that, in general, it may be difficult to incorporate down- weighting into existing clustering algorithms that are tailored for cytometry data, but indeed it is worth considering.
  One of the easy solutions to ensure that each sample contributes equally to the outcome could be down-sampling so that equal amount of cells from each sample is used in clustering. However, there are two main drawbacks of this strategy. First, a substantial amount of data (cells) may be removed from the analysis resulting in information loss. Second, during down-sampling, some of the rarer populations may become underrepresented or even skipped. Overall, it is also hard to know exactly what "drive the clustering" really means. We highlight these issues now in the "Discussion" section.
2. NRS can be used to define new panels as it was done in Levine et al. [1]. Indeed, when there is no need for redefining the panel, it can be used as a quality control step. We mention that now in the "Marker ranking based on the non-redundancy score" section.
  In Levine et al., the NRS score was used to identify a set of surface markers that is "sufficient" to detect the main clusters in the AML data. As the number of markers that can be measured is limited, they could use only 16 channels for surface markers, while the remaining 15 channels were used for signaling markers. Based on average NRS, they identified the 16 surface markers, among 42, that explained the highest amount of variance in their data. The final set was slightly redefined (two markers with high scores were excluded and two markers with low scores were included) based on the biological knowledge, which agrees with the reviewer’ statement that low variance contributions in PCA do not mean that the marker is not relevant and vice versa. However, we think that NRS can still serve as a relevant guide in marker selection. The final set was then used in the panels in the following experiments.
3. In general, as rule of thumb, we would suggest setting the number of consensus clusters (parameter maxK in ConsensusClusterPlus) to at least twice the number of expected cell populations. As this number increases, it is necessary to also increase the size of the grid in the SOM step (parameters xdim and ydim in BuildSOM).  It is not necessary to know the exact number of cell types but rather the upper boundary for this number and treat that as the expected number. We also propose a strategy of re-clustering, where first main cell types are identified and extracted and then reclustered in a secondary analysis.  In the updated version of our workflow, we have also added a section called "Obtaining higher resolution" where we describe solutions using a higher amount of clusters for higher resolution.
4. Yes, we fully agree that including batch effects into the linear modeling does not fully protect the clustering from the batch effect. That is why it is important to account for batches already at the clustering step if possible, although this is itself still a rather open (methodological research) question. Current approaches rely on, for example, equalizing the dynamic range between batches for each marker (e.g. normalization to the 0-1 range, z-scores, quantile normalization), the use of warping functions to eliminate non-linear distortions (cydar [2]), or learning marker distribution shifts between the batches based on a manually gated reference cell type and using it to correct marker expression for the whole dataset (CellCnn [3]). A recent method called MASC [4] that, similarly to our workflow, employs mixed models for the differential abundance analysis deals with batches by identifying and excluding from the clustering analysis markers with high between-batch variability and poorly recorded cells, such as cells with extreme expression values. One could also consider, batch-wise clustering and aggregation, but these strategies also require further study. However, the effectiveness of these approaches has not been sufficiently studied, yet. We still recommend including batch information in the differential analysis as it may further help to mitigate the problem.
5. Indeed, we agree that the size of clusters should be built into in the inference. This happens automatically in the differential abundance analysis, since we use (over-dispersed) logistic regression. In the differential marker expression analysis, where the medians are compared, one could account for the variability of medians calculated over clusters by assigning lower weights to clusters with lower cell counts. We have not done this in the current workflow, as we are still assessing the effect of it on the power and error control of the methods.

MINOR POINTS:

- We now mention that the fsApply function, by default, combines intensity matrices from all data sets.
- We have now added some text explaining that the glht function uses t-tests to test the hypothesis.
- We have now updated the figure captions, especially those that correspond to Figures 4, 6, 7, 8, 14, 28, 30, and 32.
- We have fixed the identified typos.
- We have updated the description of the PBMC dataset.

References

 [1]  Jacob H. Levine, Erin F. Simonds, Sean C. Bendall, Kara L. Davis, El-ad D. Amir, Michelle D. Tadmor, Oren Litvin, Harris G. Fienberg, Astraea Jager, Eli R. Zunder, Rachel Finck, Amanda L. Gedman, Ina Radtke, James R. Downing, Dana Pe’er, and Garry P. Nolan. *Data-Driven Phenotypic Dissection of AML Reveals Progenitor-like Cells that Correlate with Prognosis*. **Cell**, 162(1):184–97, jun 2015.

 [2]  Aaron T L Lun, Arianne C Richard, and John C Marioni. *Testing for differential abundance in mass cytometry data*. **Nat Meth**, 14(7):707–709, jul 2017.

 [3]  Eirini Arvaniti and Manfred Claassen. *Sensitive detection of rare disease-associated cell subsets via representation learning*. **Nature Communications**, 8:14825, apr 2017.

 [4]  Chamith Y Fonseka, Deepak A Rao, Nikola C Teslovich, Susan K Hannes, Kamil Slowikowski, Michael F Gurish, Laura T Donlin, Michael E Weinblatt, Elena M Massarotti, Jonathan S Coblyn, Simon M Helf- gott, Derrick J Todd, Vivian P Bykerk, Elizabeth W Karlson, Joerg Ermann, Yvonne C Lee, Michael B Brenner, and Soumya Raychaudhuri. *Reverse Association Of Single Cells To Rheumatoid Arthritis Accounting For Mixed Effects Identifies An Expanded CD27- HLA-DR+ Effector Memory CD4+ T Cell Population*. **bioRxiv**, 2017.
